# Constrained Phosphine Chalcogenide Selenoethers Supported by *peri*-Substitution

**DOI:** 10.3390/molecules28217297

**Published:** 2023-10-27

**Authors:** Anna E. Tarcza, Alexandra M. Z. Slawin, Cameron L. Carpenter-Warren, Michael Bühl, Petr Kilian, Brian A. Chalmers

**Affiliations:** EaStCHEM School of Chemistry, University of St Andrews, North Haugh, St Andrews, Fife KY16 9ST, UK

**Keywords:** *peri*-substitution, selenium, phosphorus, NMR, single-crystal X-ray structures, rotational conformation, DFT calculations

## Abstract

A series of phosphorus and selenium *peri*-substituted acenaphthene species with the phosphino group oxidized by O, S, and Se has been isolated and fully characterized, including by single-crystal X-ray diffraction. The P(V) and Se(II) systems showed fluxional behavior in solution due to the presence of two major rotamers, as evidenced with solution NMR spectroscopy. Using Variable-Temperature NMR (VT NMR) and supported by DFT (Density Functional Theory) calculations and solid-state NMR, the major rotamers in the solid and in solution were identified. All compounds showed a loss of the through-space *J*_PSe_ coupling observed in the unoxidized P(III) and Se(II) systems due to the sequestration of the lone pair of the phosphine, which has been previously identified as the major contributor to the coupling pathway.

## 1. Dedication

This paper is dedicated to Professor J. Derek Woollins on the occasion of his well-earned retirement and for his outstanding contributions to main group chemistry.

## 2. Introduction

**Figure 1:**
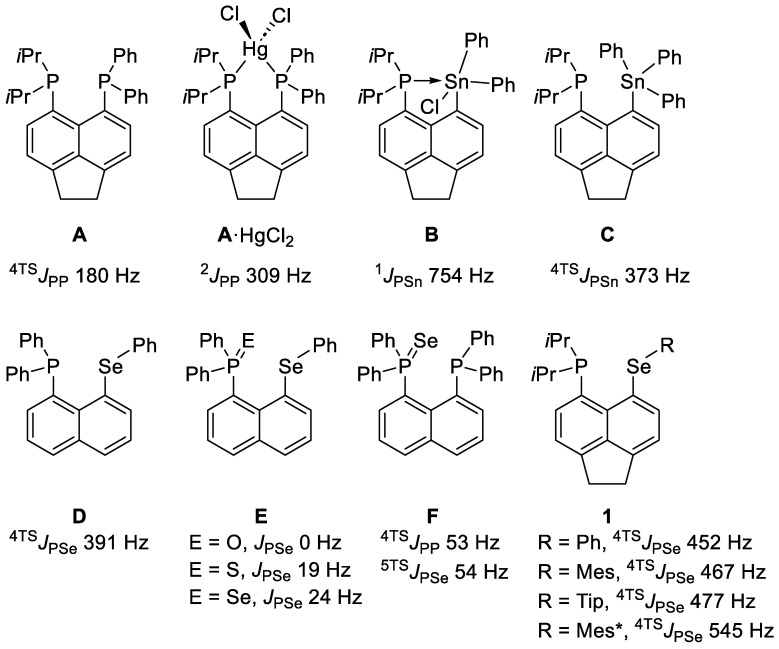
Compounds discussed in the introduction with selected *J* couplings shown (Mes = 2,4,6-trimethylphenyl; Tip = 2,4,5-triisopropylphenyl; Mes* = 2,4,6-tri-tert-butylphenyl).

The selective, stepwise lithiation reaction of 5,6-dibromoacenaphthene allows synthetic access to heteroleptic bis(phosphino)acenaphthenes and has been used to synthesize bis(phosphine) **A** (Figure 1) [1]. Due to the inherent asymmetry of the heteroleptic phosphine groups, in the ^31^P{^1^H} NMR spectrum, **A** shows two doublets of an AB spin system at δ_P_ −11.3 and −12.8 ppm, with a remarkably large ^4TS^*J*_PP_ of 180.0 Hz. This is attributed to the through-space coupling resulting from the overlap of the phosphorus lone pairs due to the constraints imposed by the rigid acenaphthene skeleton. Oxidation of the P(III) centers to P(V) with sulfur, atmospheric oxygen, or hydrogen peroxide results in a loss or significant decrease in the magnitude of the through-space *J*_PP_ coupling as the lone pairs of the phosphines are sequestered [1,2]. In only a handful of cases, where **A** acts as a bidentate ligand with MCl_2_ (M = Zn, Cd, Hg), the magnitude of *J*_PP_ increases as the coupling is mediated by the large, diffuse *s*-character orbitals of the group 12 metals (e.g., **A**·HgCl_2_ *J*_PP_ 309 Hz, Figure 1) [2].

Heteroleptic substitution is not limited to phosphorus substituents but can also involve other *p*-block and *d*-block heteroatoms (for some examples, see references [3,4,5,6,7,8,9,10,11]). Not only do heteroatoms present a challenging synthetic opportunity for *peri*-substitution, but they yield interesting NMR spectra when both nuclei are NMR active, as these nuclei can also experience through-space spin–spin coupling. When heavier nuclei are used, the orbitals are larger and more diffuse. As a result, through-space coupling can occur at longer *peri*-distances [12]. An excellent example of this is the series of phosphine–tin *peri*-substituted acenaphthene reported by Athukorala Arachchige et al. where ^31^P···^119^Sn *J* coupling can be observed [13]. The ^119^Sn isotope has *I* = ½ and a natural abundance of 8.6%, making it possible to observe *J* coupling with ^31^P (*I* = ½, 100%). In **B** (Figure 1), there is a direct P–Sn bond (2.815(3) Å) with ^1^*J*_PSn_ 754 Hz, yet, in **C**, where there is no direct P–Sn bond but there is a sub-van der Waals P···Sn interaction (3.251(1) Å), a significant *J*_PSn_ of 373 Hz is still observed, demonstrating a clear 3c–4e type overlap of the phosphorus lone pair with the Sn–C_Ph_ σ* orbital. Other P/Sn acenaphthenes have also been reported with diphenylphosphino groups instead of diisopropylphosphino groups [14].

Woollins et al. previously published a series of naphthalene-based phosphine selenoethers [15]. In **D**, there is an efficient transfer of spin information between P and Se, as indicated by the ^4TS^*J*_PSe_ of 391 Hz (note, TS superscript indicates through-space coupling). When the P(III) center is oxidized with chalcogens to P(V) (compounds **E**), the magnitude of *J*_PSe_ diminishes to <24 Hz (Figure 1) [15]. An in-depth computational study has shown that the magnitude of *J*_PP_ and *J*_PSe_ in the related compound **F** has contributions from both through-space and through-bond pathways [16]. We recently reported a series of acenaphthene analogues (**1**) with various aryl groups bound to selenium [17]. As the electron-donating ability of the aryl group attached to selenium increases, so does the magnitude of *J*_PSe_ from 452 Hz, when R = phenyl, up to 545 Hz, when R = Mes* (2,4,6-tri-*tert*-butylphenyl).

## 3. Results and Discussion

### 3.1. Synthesis

**Scheme 1:**
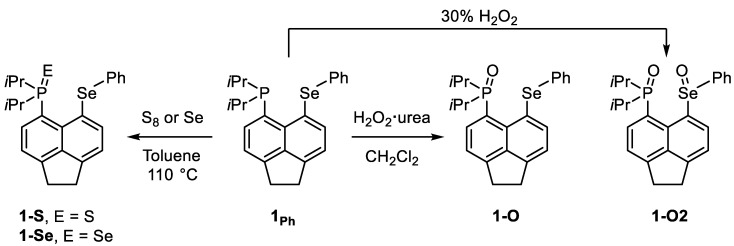
The synthetic pathway for compounds **1-O**, **1-S**, **1-Se**, and **1-O2**.

Utilizing compound **1_Ph_** as our workhorse, we herein report the synthesis and characterization of the P(V) chalcogen oxidized species **1-O**, **1-S**, and **1-Se** and the P(V)/Se(IV) species **1-O2** (Scheme 1).

Compound **1_Ph_** showed a singlet in the ^31^P{^1^H} NMR spectrum at δ_P_ −6.5 ppm with ^77^Se satellites giving *J*_PSe_ 452.2 Hz. This was complemented by a doublet at δ_Se_ 425.3 ppm, observed in the ^77^Se{1H} NMR spectrum. Heating a solution of **1_Ph_** under reflux in toluene with one equivalent of gray selenium for 15 h, followed by purification, afforded **1-Se** as a yellow microcrystalline powder (54% yield). **1-S** was prepared in a similar manner but only required heating under reflux for 6 h for complete consumption of **1_Ph_**. After purification, **1-S** was afforded as beige microcrystalline powder (86% yield).

The first attempt to produce **1-O** using hydrogen peroxide resulted in the formation of a mixture of **1-O** and **1-O2**, as determined by solution ^77^Se{^1^H} NMR spectroscopy. Direct synthesis of **1-O2** was achieved by using an excess of hydrogen peroxide. After purification, **1-O2** was obtained as a white solid (45% yield). To control the oxidation of **1_Ph_** to selectively oxidize the phosphine, we attempted air oxidation by leaving a vigorously stirring solution of **1_Ph_** exposed to air; however, even after 24 h, no reaction had occurred, as judged by ^31^P{^1^H} NMR spectroscopy. Instead, **1_Ph_** was stirred with one equivalent of H_2_O_2_·urea complex. The conversion was slow, but, as monitored by ^31^P{^1^H} NMR spectroscopy, complete consumption of **1_Ph_** was observed after 72 h. After recrystallization, **1-O** was isolated in a 39% yield. The mechanism of the phosphine oxidation was not studied in the scope of this work, as P(III) to P(V) oxidations by peroxides, cyclooctasufur (S_8_), and gray selenium are well established from early thermochemical and mechanistic studies [18,19,20]. All compounds reported herein were found to be air stable, in the solid state, with no signs of degradation after twelve months.

### 3.2. Crystallography

**Figure 2:**
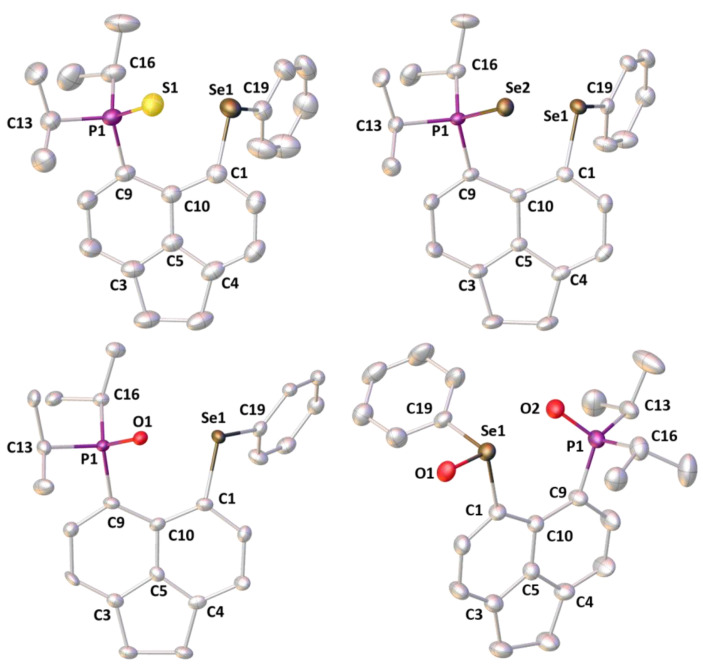
Molecular structures of **1-S**, **1-Se**, **1-O**, and **1-O2**. Hydrogen atoms and the second molecule in the asymmetric unit of **1-O2** are omitted for clarity. Anisotropic displacement ellipsoids are plotted at the 50% probability level.

Crystals of **1-Se**, **1-S**, and **1-O** were grown from a solution of CH_2_Cl_2_:hexane (1:3 *v*/*v*), and crystals of **1-O2** were grown from evaporation of a solution in CH_2_Cl_2_. The structures of **1-O**, **1-S**, and **1-Se** are very similar with only minor differences due to the increased size of the chalcogen bound to phosphorus. The crystal structures are shown in Figure 2, and selected crystallographic data are presented in Table 1.

The most notable differences between the structures of the precursor (**1_Ph_**) [17] and the oxidized P(V) species (**1-O**, **1-S**, and **1-Se**) are in the *peri*-region. In **1_Ph_**, the P···Se distance is 3.055(1) Å; this increases to 3.322(2) Å in **1-O**, 3.4863(5) Å in **1-S**, and 3.5012(7) Å in **1-Se**. Similarly, there are large increases in the splay angles (12.6° in **1_Ph_** to 32.0° in **1-Se**), the P–C···C–Se dihedral angles, and the out-of-plane displacements of the P and Se atoms from the mean C_12_ acenaphthene plane (see Table 1). These changes are expected due to the new steric demands placed on the molecule caused by the addition of another atom into the *peri*-gap when the *i*Pr_2_P group is oxidized to *i*Pr_2_P = E (where E = O, S, Se). When compared to the crystal structures of the naphthalene analogues (**E**, Figure 1), there are no significant differences [15]. The only dissimilarity observed between the acenaphthene and naphthalene analogues is that the absence of the ethylene bridge in the naphthalene structures results in a slightly decreased P···Se distance and slightly smaller splay angles. For example, in the naphthalene compound **E** (where E = Se), the P···Se distance is 3.278(2) Å (vs. 3.5012(7) Å for **1-Se**), and the splay angle drops from 32.0° to 24.8°.

The structure of **1-O2** is very different from that of the other three due to the Se(IV) group (selenoxide) being present. The trends are similar to those observed in the Se(II) complexes, but taken to a new extreme. There is a greater steric demand on the molecule, as evidenced by the much larger splay angle of 28° (29° in the second molecule of the asymmetric unit) and *peri*-distance between the P and Se atoms of 3.578(1) Å (3.610(1) Å). Somewhat unexpectedly, the dihedral angle is much smaller at 1.8(3)° (1.7(3)°); however, this arises from the rotation around the C9–Se1 bond, such that the Se=O group points away from the *peri*-gap, which significantly reduces the steric crowding and the need for any out-of-plane deformations to relieve the steric strain.

### 3.3. NMR Spectroscopy of **1-Se**

In solution, the precursor **1_Ph_** showed a sharp singlet in the ^31^P{^1^H} NMR spectrum at δ_P_ −6.5 ppm with ^77^Se satellites (7.6% natural abundance), giving ^4TS^*J*_PSe_ of 452.2 Hz. This was complemented by a doublet in the ^77^Se{^1^H} NMR spectrum centered at δ_Se_ 425.3 ppm (^4TS^*J*_SeP_ 452.8 Hz). Recently, we have shown that the large through-space coupling between ^31^P and ^77^Se arises from the overlap of the phosphorus lone pair with the orbitals localized on the Se–C_Ph_ bond [17]. In this study, the lone pair of the phosphorus was sequestered by oxidation with a chalcogen atom in all complexes, and as expected, this significantly reduced the magnitude of *J*_PSe_ in all compounds. Based on the recent findings by Makina et al. [16], we assume the dominant pathway of coupling information being exchanged is through space in the unoxidized **1_Ph_**; therefore, we attribute the drop in magnitude to *J*_PSe_ to the loss of this pathway.

#### 3.3.1. Fluxionality in Solution

**Figure 3:**
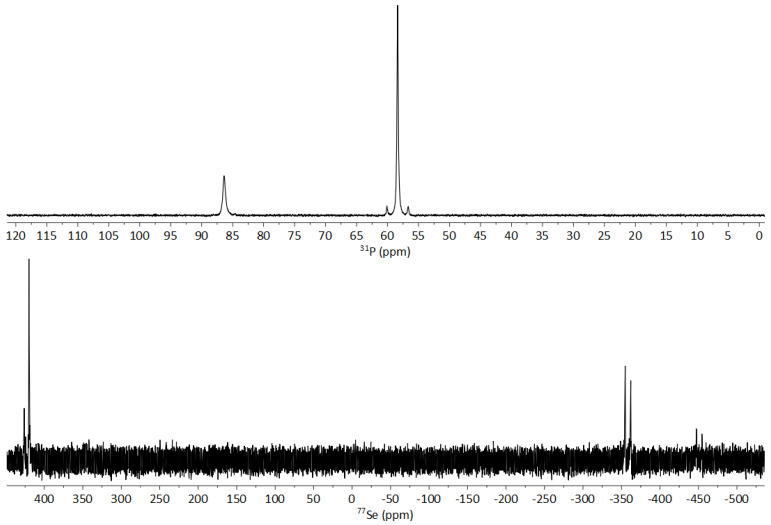
The ambient temperature ^31^P{^1^H} (**top**) and ^77^Se{^1^H} (**bottom**) NMR spectra of **1-Se**, acquired at 202.5 and 95.4 MHz, respectively.

The ^31^P{^1^H} NMR spectrum of **1-Se** revealed two singlets, at δ_P_ 86.3 and 58.4 ppm, when only one was anticipated (Figure 3, top). The signal at δ_P_ 58.4 ppm was accompanied by a broadened set of ^77^Se satellites with ^1^*J*_PSe_ of ca. 690 Hz. This was indicative of a P = Se double bond and closely resembled those reported in the literature (cf. Ph_3_PSe; ^1^*J*_PSe_ 730 Hz) [21]. The signal at δ_P_ 86.3 ppm showed significant broadening and no resolvable ^77^Se satellites. The presence of two rotational conformers was confirmed by ^77^Se{^1^H} NMR with the spectrum showing two broad singlets at δ_Se_ 426.2 and 419.0 ppm corresponding to the selenoether, as well as two doublets at δ_Se_ −358.5 and −451.0 ppm (^1^*J*_SeP_ ca. 696 and 693 Hz, respectively) corresponding to the phosphine selenide (Figure 3, bottom). Initially, the presence of two sets of peaks suggested the occurrence of a side reaction; however, upon further investigation using Variable-Temperature NMR, it was concluded they were due to the very large steric bulk around the *peri*-region exhibiting fluxional behavior in solution. Acquisition of a ^1^H–^31^P HMBC spectrum (Figure S1) showed a strong correlation between both ^31^P signals and the hydrogen atoms in the isopropyl groups, strongly supporting the idea of fluxional behavior. This was unexpected as the naphthalene equivalent (**E**) was reported as showing one sharp singlet in the ^31^P{^1^H} NMR spectrum, which does not suggest fluxional behavior [15].

To obtain further evidence of the fluxional behavior, a one-dimensional exchange spectroscopy (EXSY) NMR experiment was performed on **1-Se**. This showed magnetization transfer within the NMR timescale at 253 K. This indicated an exchange between two magnetic environments in two different isomers and confirmed the presence of fluxional behavior in solution. Therefore, the two sets of signals observed in the ambient temperature (293 K) ^31^P{^1^H} and ^77^Se{^1^H} NMR spectra arose from two different rotational conformations present in solution. The notion of different rotational conformations in solution in *peri*-substituted naphthalenes has been reported by Woollins previously [22]. The species Nap(POCl_2_)(PCl_2_) (Nap = naphthalene-1,8-diyl), with a P(III)/P(V) *peri*-substitution, was demonstrated to have two rotamers in solution, with the ^31^P{^1^H} NMR spectrum at 233 K showing two similar signals for the PCl_2_ group (δ_P_ 145.52 and 145.50 ppm) and one signal for the POCl_2_ group (δ_P_ 42.9 ppm). At 298 K, the signals at δ_P_ 145.52 and 145.50 ppm were not observed. Kilian et al. reported that these results are interpreted as “the hindered rotation around the P-C_(Nap)_ bonds, resulting the presence of two conformers whose interconversion is slow on the NMR time scale”.

**Figure 4:**
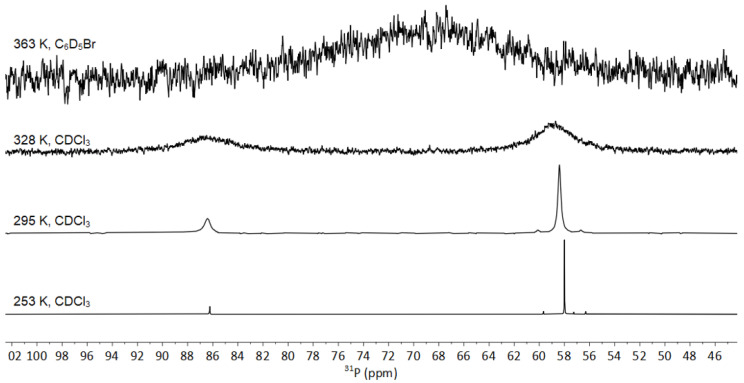
The ^31^P{^1^H} VT NMR spectra of **1-Se** with solvent and temperatures indicated (acquired at 202.5 MHz).

**Figure 5:**
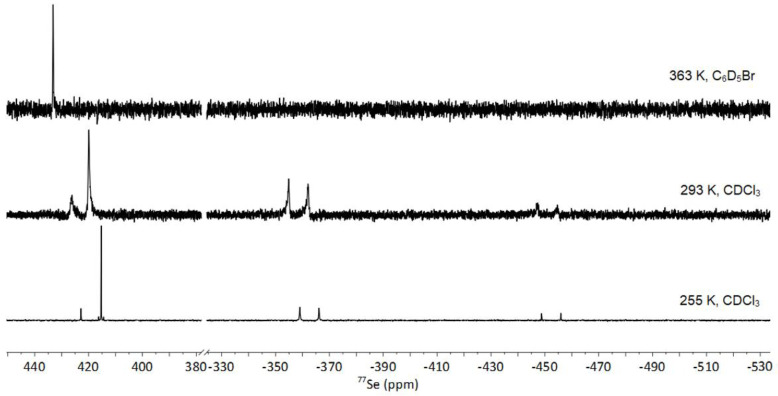
The ^77^Se{^1^H} VT NMR spectra of **1-Se** with solvent and temperatures indicated (acquired at 95.4 MHz).

Variable-temperature ^31^P{^1^H} and ^77^Se{^1^H} NMR experiments were carried out using **1-Se**; however, to overcome the coalescence point, a high-boiling solvent was needed. For the elevated-temperature experiments, d_5_-bromobenzene (boiling point 156 °C, 429 K) was used as the NMR solvent. For low-temperature experiments, d-chloroform (melting point −64 °C, 209 K) was used. At 253 K, fully resolved signals of the two conformations with observable satellites were observed. The ^31^P{^1^H} NMR spectrum at 253 K showed two singlets at δ_P_ 86.2 and 58.0 ppm, with ^1^*J*_PSe_ of 682.9 and 681.3 Hz, respectively (Figure 4). The ^77^Se{^1^H} NMR spectrum at 255 K showed two singlets at δ_Se_ 422.8 and 415.3 ppm corresponding to the selenoether and two doublets at δ_Se_, −362.6 and −452.4 ppm, with ^1^*J*_SeP_ 681.5 and 683.6 Hz, corresponding to the phosphine selenide (Figure 5). The singlet at δ_Se_ 415.3 ppm also showed a ^5TS^*J*_SeSe_ coupling of 182.0 Hz as ^77^Se satellites (Figure 5). Even in the slow-motion regime at 253–255 K, no through-space coupling was present between the phosphorus and the selenoether. This was expected as the phosphorus lone pair was sequestered in the P = Se bond and, hence, was no longer available to overlap with the SePh orbitals. The ^31^P{^1^H} NMR spectrum acquired at 363 K showed one broad singlet at δ_P_ 68.3 ppm as the energy barrier between the two conformations had been overcome, but the speed of the exchange was only marginally faster than the NMR timescale. At 363 K, the ^77^Se{^1^H} NMR spectrum showed one singlet at δ_Se_ 433.0 ppm, corresponding to the selenoether. The upfield signal attributed to the P = Se group was not observed, likely due to the fact that 363 K is close to the coalescence temperature. Due to limitations with the equipment, we could not acquire any data at temperatures exceeding 368 K.

As the coalescence was observed in the ^31^P{^1^H} and the ^77^Se{^1^H} NMR spectra, the coalescence method could be used to estimate the rotational barrier (Δ*G*^‡^) of **1-Se**, assuming the coalescence followed typical Eyring behavior.

$$\Delta G^{‡}=aT_{C}\left[9.972+log\left(\frac{T_{c}}{\Delta \nu }\right)\right]$$

Using this equation, the temperature of coalescence (*T_C_* = 363 K) and the largest separation between the signals of the two conformers obtained from the lowest-temperature ^31^P{^1^H} VT NMR spectra (Δ*ν* = 5715 Hz); Δ*G*^‡^ was estimated as 61 kJ mol^−1^.

#### 3.3.2. Solid-State NMR of **1-Se**

**Figure 6:**
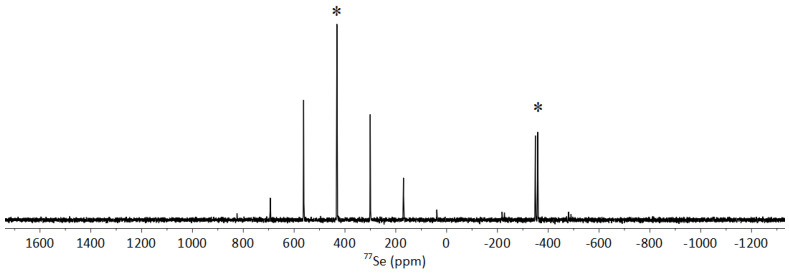
The ^77^Se{^1^H} SS-MAS NMR spectrum of **1-Se** recorded at 76.3 MHz. The isotropic peaks are located at δ_Se_ 431.6 and −353.9 ppm and denoted with *.

To corroborate the large coupling values observed and to confirm the number of conformations in the solid state, ^31^P{^1^H} (Figure S2) and ^77^Se{^1^H} SS-MAS NMR spectra of **1-Se** were acquired (Figure 6). In the ^31^P{^1^H} SS-MAS NMR spectrum, a singlet at δ_P_ 60.2 (with ^77^Se satellites giving ^1^*J*_PSe_ = 699.2 Hz) was observed, with spinning sidebands. In the ^77^Se{^1^H} SS-MAS NMR spectrum, there were two signals: a singlet at δ_Se_ 431.6 ppm and a doublet at δ_Se_ −353.9 ppm, with ^1^*J*_SeP_ of 700.8 Hz (Figure 6). The singlet corresponds to the selenoether and the doublet to the phosphine selenide environment. The large ^1^*J*_SeP_ was still present in the solid state, albeit with a slightly larger magnitude than in the solution state.

The key finding is that no other signals were present for each ^31^P and ^77^Se environment, indicating that while the bonding environments and connections were the same in both the solution and the solid state, only one conformer was present in the solid state. If two conformers were present in the solid state, two isotropic signals (with spinning side bands) would be expected in both the upfield and downfield regions of the ^77^Se{^1^H} MAS spectrum. It is likely that the dominant isomer corresponded to the conformation elucidated by the crystal structure (Figure 2). However, it is also possible that some solvates were formed, as demonstrated recently [23].

### 3.4. NMR Spectroscopy of **1-S** and **1-O**

For other chalcogen-oxidized compounds of **1_Ph_**, the change in rotational barrier was expected to follow the trend **1-Se** > **1-S** > **1-O**, as the larger atomic radius of selenium provides a greater barrier to the rotation of the molecule (single-bond covalent radii Se 1.16 Å; S 1.03 Å; O 0.63 Å) [24].

**Figure 7:**
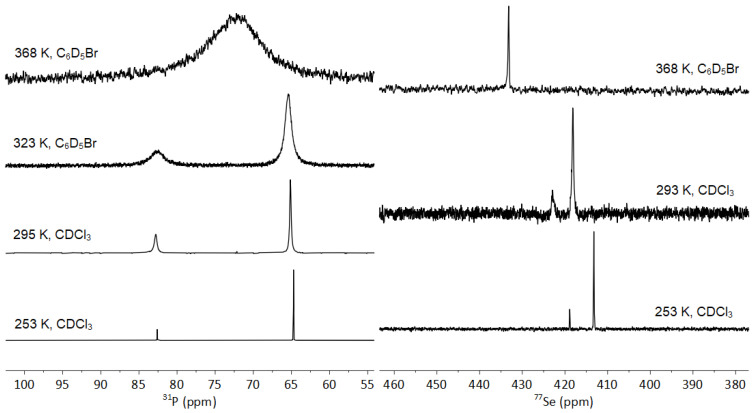
The ^31^P{^1^H} VT NMR spectra (**left**) and ^77^Se{^1^H} VT NMR spectra (**right**) of **1-S** with solvent and temperatures indicated (acquired at 202.5 and 95.4 MHz, respectively).

The lighter congeners, **1-S** and **1-O** were prepared; as with **1-Se**, the solution state ^31^P{^1^H} NMR spectrum of **1-S** acquired at ambient conditions was notably broad with two signals at δ_P_ 82.8 and 65.1 ppm, neither of which showed any ^77^Se satellites (Figure 7, left). The solution-state ^77^Se{^1^H} NMR spectrum mirrored the observations of the ^31^P{^1^H} spectrum with two broad singlets present at δ_Se_ 422.9 and 418.2 ppm (Figure 7, right). To determine the coalescence temperature and thus determine the rotational energy barrier, VT NMR experiments were performed on **1-S** (Figure 7). The ^31^P{^1^H} NMR spectra showed coalescence was reached at 368 K, although the signal at δ_P_ 71.9 ppm was still observed as a reasonably broad singlet, while completely resolved signals of the two rotamers in the slow-motion regime were observed at 253 K, showing two singlets at δ_P_ 82.6 and 64.7 ppm. Comparatively, the ^77^Se{^1^H} NMR spectra showed fast free rotation was achieved at 368 K with a sharp singlet observed at δSe 433.2 ppm and verified the full resolution of signals in the slow-motion regime at 255 K (δ_Se_ 418.9 and 413.2 ppm).

Similar observations were made for **1-O**. The ^31^P{1H} NMR spectrum at ambient conditions revealed two broad singlets at δ_P_ 55.4 and 54.3 ppm, with the ^77^Se{^1^H} NMR spectrum showing two singlets at δ_Se_ 436.6 and 400.9 ppm (Figures S3 and S4). Additionally, VT NMR studies were carried out, with the coalescence observed at 323 K in the ^31^P{^1^H} NMR spectrum with complete sharpening of signals observed at 373 K. Two fully resolved singlets were observed in both the ^31^P{^1^H} and ^77^Se{^1^H} spectra at 255 K (δ_P_ 56.1 and 55.2 ppm; δ_Se_ 430.4 and 397.0 ppm). Using Δ*ν* = 3617 Hz from the 255 K spectra for **1-S** and Δ*ν* = 184 Hz from the 255 K spectra for **1-O**, Δ*G*^‡^ was estimated to be ca. 62 kJ mol^−1^ for **1-S** and 63 kJ mol^−1^ for **1-O** (at 323 K), which, somehow contrary to expectations, was marginally higher than for **1-Se** (61 kJ mol^−1^). However, one needs to realise that the coalescence method is only an approximation, and that the Δ*G*^‡^ values were obtained for different temperatures. Full Erying analysis was not possible as the spectrometer could not exceed 373 K, meaning complete sharpening of the peaks was never observed. As the van der Waals radii of Se is larger than that of S, which is larger than that of O (1.93, 1.85, 1.37 Å, respectively) [25], one may expect the rotational barriers to follow this order; therefore, it is likely that other steric and electronic effects were dominant here.

### 3.5. NMR Spectroscopy of **1-O2**

The solution-state ^31^P{^1^H} NMR spectrum of **1-O2** showed one sharp downfield-shifted singlet at δ_P_ 56.4 ppm with the ^77^Se{^1^H} NMR spectrum also showing a sharp downfield-shifted singlet at δ_Se_ 896.4 ppm (cf. **1_Ph_** δ_P_ −6.5 ppm; δ_Se_ 425.3 ppm) (Figure S5). The large downfield shift of both peaks was consistent with the oxidation of both the *i*Pr_2_P and SePh moieties to the P(V) and Se(IV) species, *i*Pr_2_P(O) and Se(O)Ph. No other signals were present in the NMR spectra, unlike for **1-O**, **1-S**, and **1-Se**, indicating that only one rotational conformation was present in solution, presumably due to the increased steric bulk in the *peri*-region causing extremely hindered rotation of the *i*Pr_2_P(O) and Se(O)Ph groups. Due to the phosphorus lone pair being sequestered, as well as one of the selenium lone pairs, no *J*_PSe_ couplings were observed in either spectra.

### 3.6. Computational Studies

**Figure 8:**
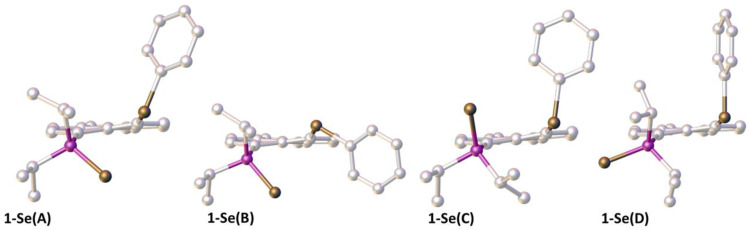
B3YP-D3 optimized rotamers of **1-Se** viewed along the central C–C bond of the acenaphthene moiety; 1-Se(A) is the conformation observed in the solid state. Color code: gray, purple, and bronze for C, P, and Se, respectively. Hydrogen atoms are omitted for clarity.

To complement these findings, we performed calculations at the B3LYP-D3/6-311+G(d,p)/CPCM(C_6_H_5_Br)//B3LYP-D3/6-31+G(d,p) level of density functional theory (DFT). Starting from the conformation observed in the solid, selected rotamers were constructed by rotating the SePh and *i*Pr_2_P(Se) moieties about the C(acenaphthene)–E bonds (E = P, Se). The resulting optimized structures are shown in Figure 8, and computed relative energies are collected in Table 2.

In the conformer found in the solid (structure **1-Se(A)** in Figure 8), the two Se atoms displayed sub-van der Waals contact (Se···Se distance 3.29 Å and 3.28 Å from B3LYP-D3 and XRD, respectively), and the Se–Ph group was oriented along the Se···Se axis and anti with respect to the Se atom on the phosphine. Rotating either the SePh group or the *i*Pr_2_P(Se) group such that the Se atoms were still in contact but the SePh group was roughly perpendicular to the Se···Se axis afforded two minima (**1-Se(B)** and **1-Se(C)**, respectively, in Figure 8) which were significantly higher in energy than conformer **1-Se(A)** (by ca. 19–34 kJ mol^−1^, see Table 2). Further rotating the *i*Pr_2_P(Se) moiety such that the Se atom on the phosphine was pointing away from the other Se atom in the SePh substituent afforded a new minimum (**1-Se(D)** in Figure 8) which was slightly more stable than conformer **1-Se(A)** (by ca. −2 to −3 kJ mol^−1^, see Table 2). One of the isopropyl groups was also rotated to minimize steric clash between a methyl group and the Se(Ph) atom in rotamer **1-Se(D)**. These results are fully compatible with the observation of a mixture of two slowly interconverting isomers. Based on the comparison of computed and observed ^31^P and ^77^Se chemical shifts (see Table 2), we assigned the more deshielded ^31^P resonance, and the more “extreme” ^77^Se shifts (i.e., the most deshielded and the most shielded one), to rotamer **1-Se(D)**. From the observed relative intensities of these two sets of signals (Figure 4 and Figure 5), it appears that it was indeed rotamer **1-Se(A)** that was more abundant, i.e., more stable. This assignment also agrees with the comparison of the ^77^Se resonances observed in the solid (Figure 6), arguable arising from **1-Se(A)**, and those of the more abundant form in solution. In addition, only for **1-Se(A)**, a notable indirect *J*_SeSe_ spin–spin coupling constant was computed (145 Hz, with 182 Hz observed), whereas that in **1-Se(D)** was negligibly small. The reason why the computed relative stabilities of **1-Se(A)** and **1-Se(D)** were reversed is not clear at the moment. Indeed, switching the solvent model to CHCl_3_, or the functional to M06-2X, which has performed very well for energetics in other related systems [5], did not change the relative sequence of both.

The reason for the apparent stability of rotamer **1-Se(D)** seems to be more the relief of Se···Se repulsion rather than Se···P bonding interactions; the optimized Se···P distance in **1-Se(D)** was 3.72 Å. This is close to the sum of the van der Waals radii of 4.09 Å; consequently, only a very small Wiberg bond index of 0.01 was obtained between these two atoms.

## 4. Materials and Methods

### 4.1. General Considerations

All synthetic manipulations were performed under an atmosphere of dry nitrogen using standard Schlenk techniques or under an argon atmosphere in a Saffron glove box. However, all compounds reported herein were found to be air stable, so repeated reactions were performed under air with no detrimental effects. All glass apparatus were stored in a drying oven (ca. 120 °C) prior to use. Dry solvents were collected from an MBraun Solvent Purification System and stored over appropriate molecular sieves. Water used in experiments was subject to nitrogen sparging and stored under nitrogen prior to use. Chemicals were taken from the laboratory inventory and used without further purification. Infrared Spectra were acquired using a Nicolet 308 FT-IR (Thermo Fisher Scientific, Oxford, UK) with Specac ATR attachment, recorded between 4000 and 500 cm^−1^.

**Figure 9:**
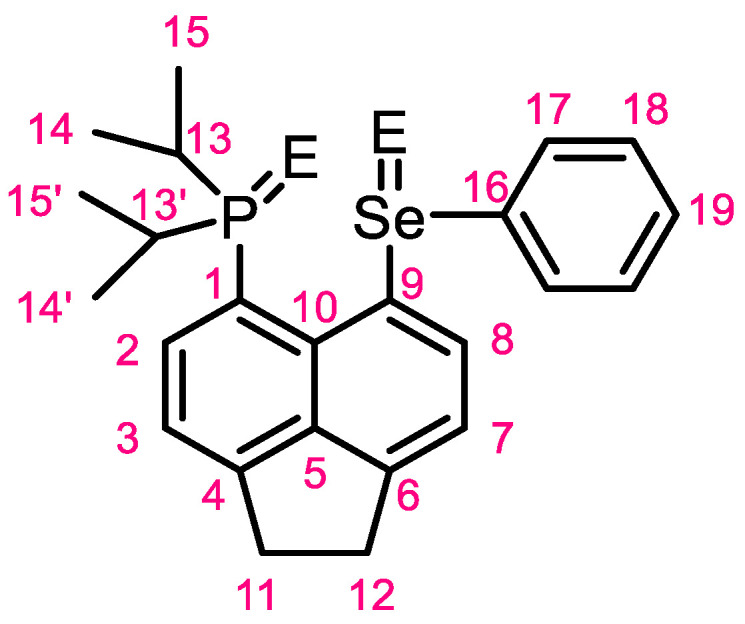
NMR numbering system for compounds reported (E = null, O, S, Se).

All solution-state NMR spectra were recorded using either a Bruker Avance III (500 MHz) or Bruker Avance III-HD (500 MHz) spectrometer operating at a magnetic field strength of 11.7 Tesla at 20 °C, unless otherwise specified. Assignments of ^1^H and ^13^C spectra were made in conjunction with appropriate 2D spectra. ^13^C NMR spectra were recorded using the DEPTQ pulse sequence with broadband proton decoupling. The following external standards were used: ^1^H and ^13^C NMR, tetramethylsilane; ^31^P NMR, 85% H_3_PO_4_ in D_2_O; ^77^Se NMR, dimethyldiselende (Me_2_Se_2_) and diphenyldiselenide (Ph_2_Se_2_) as a secondary reference at 463.0 ppm. Residual solvent peaks were also used for secondary calibration (CDCl_3_ δ_H_ 7.260 ppm; δ_C_ 77.160 ppm; C_6_D_5_Br δ_H_ 7.300, 7.019, 6.946 ppm; δ_C_ 130.900, 129.339, 126.162, 122.181 ppm). Chemical shifts (δ) are given in parts per million (ppm) relative to the residual solvent peaks where possible. Coupling constants (*J*) are quoted in Hertz (Hz). The NMR numbering scheme for all compounds is shown in Figure 9.

Solid-state ^31^P{^1^H} and ^77^Se{^1^H} NMR (SS-MAS NMR) measurements were performed using a Bruker Avance III 400 MHz spectrometer operating at a magnetic field strength of 9.4 T. Experiments were carried out using a conventional 4 mm MAS probe with a MAS rate of 14 KHz for ^31^P{^1^H} and 10 kHz for ^77^Se{^1^H}. The ^77^Se{^1^H} cross-polarization MAS experiments (using ramped contact pulse durations of 5 ms and TPPM 1H decoupling) were carried out with signal averaging for 2048 transients with a recycle interval of 3 s. Chemical shifts are reported in ppm, relative to Me_2_Se at 0 ppm, using the isotropic resonance of solid H_2_SeO_3_ at 1288.1 ppm as a secondary reference. The position of the isotropic resonance within the spinning sideband patterns was unambiguously determined by recording a second spectrum at a different MAS rate.

Melting and decomposition points were determined by heating solid samples in sealed glass capillaries using a Stuart SMP30 Melting Point Apparatus. High-Resolution Mass Spectrometry of **1-S** and **1-Se** was performed by the EPSRC UK National Mass Spectrometry Facility (NMSF) at Swansea University using a Thermofisher LTQ Orbitrap XL (Atmospheric-Pressure Chemical Ionization). Mass Spectrometry on **1-O** and **1-O2** was performed at the University of St Andrews using a Micromass LCT (Electrospray Ionization) from solutions of the analyte in methanol or acetonitrile. Elemental Analysis was performed by the EA Service at London Metropolitan University.

### 4.2. Synthetic Procedures and Analytical Data

#### 4.2.1. Synthesis of **1-O**

A solution of **1_Ph_** (500 mg, 1.18 mmol) in dichloromethane (25 mL) was prepared. To this, a solution of hydrogen peroxide urea adduct (111 mg, 1.18 mmol) in water (100 mL) was added in one batch. The solution was stirred vigorously for three days. The organic layer was separated and dried over magnesium sulfate. The volatiles were removed in vacuo to afford the crude product. Recrystallization from dichloromethane:*n*-hexane (1:4 *v*/*v*) at −20 °C afforded analytically pure crystals of **1-O** (200 mg, 40%) (melting with decomp. 170–173 °C). These crystals were suitable for single-crystal X-ray diffraction.

**^1^H NMR:** (500.1 MHz, C_6_D_5_Br, 368 K) δ_H_ 7.84 (1H, d, ^3^*J*_HP_ 7.3 Hz, H-2), 7.20 (1H, d, ^3^*J*_HH_ 7.2 Hz, H-7), 7.19–7.13 (2H, m, H-17), 7.00–6.90 (4H, m, H-3, 18, 19), 3.15–3.03 (4H, m, H-11,12), 1.33 (6H, dd, ^3^*J*_HP_ 15.2, ^3^*J*_HH_ 6.9 Hz, H-14/15), 1.00 (6H, ^3^*J*_HP_ 15.5, ^3^*J*_HH_ 6.9 Hz, H14/15). **^13^C DEPTQ NMR:** (125.8 MHz, C_6_D_5_Br, 368 K), δ_C_ 151.1 (d, ^4^*J*_CP_ 2.4 Hz, qC-6), 147.4 (s, qC-4), 140.6 (d ^3^*J*_CP_ 8.2 Hz, qC-5), 140.3 (s, C-2), 138.2 (s, qC-16), 135.9 (d, ^2^*J*_CP_ 23.0 Hz, qC-10), 131.2 (s, C-18), 130.4 (s, qC-1/8), 128.8 (s, C-17), 126.1 (s, C-19), 125.3 (s, qC-9), 120.7 (s, C-3), 118.5 (d, ^3^*J*_CP_ 11.6 Hz, C-7), 30.0 (s, C-11/12), 29.7 (s, C-11/12), 29.5 (d, ^1^*J*_CP_ 67.0 Hz, C-13), 17.3 (d, ^2^*J*_CP_ 3.4 Hz, C-14/15), 16.9–16.8 (m, C-14/15). **^31^P{^1^H} NMR:** (202.4 MHz, CDCl_3_, 253 K) δ_P_ 56.1 (s), 55.2 (s). **^31^P{^1^H} NMR:** 202.4 MHz, CDCl_3_, 295 K) δ_P_ 55.4 (br s), 54.3 (s). **^31^P{^1^H} NMR:** (202.4 MHz, C_6_D_5_Br, 373 K) δ_P_ 53.0 (s). **^77^Se{^1^H} NMR:** (95.4 MHz, CDCl_3_, 253 K) δ_Se_ 430.4 (s), 397.0 (s). **^77^Se{^1^H} NMR:** (95.4 MHz, CDCl_3_, 295 K) δ_Se_ 436.6 (s), 400.9 (s). **^77^Se{^1^H} NMR:** (95.4 MHz, C_6_D_5_Br, 363 K) no signals observed. **IR**: ν_max_ ATR/cm^−1^ 3067w (ν_CH_), 2963w (ν_CH_), 1576m (ν_C=C_), 1138s (ν_P=O_), 851m, 733s, 691s. **HRMS:** (ES+): *m*/*z* (%) Cacld. for C_24_H_27_POSeNa: 465.0857, found: 465.0842 (100) [M+Na].

#### 4.2.2. Synthesis of **1-O2**

A solution of **1_Ph_** (500 mg, 1.18 mmol) in dichloromethane (20 mL) was prepared. To this, 30% aqueous hydrogen peroxide (0.25 mL, 2.47 mmol) was added dropwise over five minutes with vigorous stirring. The solution was stirred at ambient conditions for a further six hours. The organic layer was separated and dried over magnesium sulfate. The volatiles were removed in vacuo to afford **1-O2** as a pale orange solid (240 mg, 45%) (melting with decomp. 208–214 °C). The aqueous layer was quenched with aqueous sodium metabisulfite before disposal. Crystals of **1-O2** suitable for single-crystal X-ray diffraction were grown from a dichloromethane/*n*-hexane vapor diffusion set up at ambient conditions.

**^1^H NMR** (500.1 MHz, CDCl_3_) δ_H_ 8.45 (1H, dd, ^3^*J*_HH_ 7.5 Hz, H-8), 8.05–7.98 (2H, m, H-18), 7.57 (1H, dd, ^3^*J*_HP_ 13.7, ^3^*J*_HH_ 7.3, H-2), 7.37 (1H, d, ^3^*J*_HH_ 7.6 Hz, H-7), 7.32 (1H, d, ^3^*J*_HH_ 7.3 Hz, H-3), 7.23 (3H, m, H-17,19), 3.34–3.21 (4H, m, H-11,12), 2.54–2.36 (2H, m, H-13,13′), 1.28–1.14 (9H, m, H-14/14′/15/15′, 3 × CH_3_), 0.88 (3H, dd, ^3^*J*_HP_ 15.3, ^3^*J*_HH_ 7.1 Hz, H-14/14′/15/15′, 1 × CH_3_). **^13^C DEPTQ** (125.8 MHz, CDCl_3_) δ_C_ 152.8 (s, qC-4), 151.0 (s, qC-6), 148.3 (s, ^1^*J*_CSe_ 127.9 Hz, C-16), 140.3 (d, ^3^*J*_CP_ 8.8 Hz, C-5), 139.8 (d, ^3^*J*_CP_ 3.3 Hz, qC-9), 134.1 (d, ^2^*J*_CP_ 11.7 Hz, C-1), 132.3 (s, C-8), 132.2 (d, ^2^*J*_CP_ 4.6 Hz, qC-10), 129.5 (s, C-19), 128.5 (s, C-17), 127.7 (s, C-18), 121.5 (s, C-7), 119.2 (d, ^1^*J*_CP_ 84.8 Hz, qC-1), 118.6 (d, ^3^*J*_CP_ 13.0 Hz, C-3), 30.6 (s, C-11/12), 29.5 (s, C-11/12), 28.8 (d, ^1^*J*_CP_ 64.1 Hz, C-13/13′), 26.5 (^1^*J*_CP_ 68.3 Hz, C-13/13′), 17.1 (s, C-14/15, 1 × CH_3_), 16.1 (d, ^2^*J*_CP_ 2.1 Hz, C-14/15, 1 × CH_3_), 15.9 (d, ^2^*J*_CP_ 3.5 Hz, C-14/15, 1 × CH_3_), 15.6 (d, ^2^*J*_CP_ 1.8 Hz, C-14/15, 1 × CH_3_). **^31^P{^1^H} NMR** (202.5 MHz, CDCl_3_) δ_P_ 56.4 (s). **^77^Se{^1^H} NMR** (95.4 MHz, CDCl_3_) δ_Se_ 869.4 (s). **IR**: ν_max_ ATR/cm^−1^ 3047w (ν_CH_), 2962w (ν_CH_), 1597m (ν_C=C_), 1437m (ν_C=C_), 1149s (ν_P=O_), 818vs (ν_Se=O_), 752s, 690s. **HRMS**: (ES+): *m*/*z* (%) Cacld. for C_24_H_28_PO_2_Se: 459.0987, found: 459.0973 (100) [M+H].

#### 4.2.3. Synthesis of **1-S**

A suspension of **1_Ph_** (1.50 g, 3.52 mmol) and sulfur (177 mg, 3.65 mmol) in toluene (30 mL) was heated under reflux for six hours. The solution was cooled to ambient conditions and all volatiles removed in vacuo to afford the crude product. Recrystallization from dichloromethane:*n*-hexane (1:3 *v*/*v*) at −20 °C afforded white analytically pure crystals of **1-S** (1.38 g, 86%) (melting with decomp. 232–237 °C). These crystals were suitable for single-crystal X-ray diffraction. **Elemental Analysis**: Cacld. (%) for C_24_H_27_PSSe: C 63.01, H 5.95, found: C 62.89, H 6.03. **^1^H NMR** (500.1 MHz, C_6_D_5_Br, 363 K) δ_H_ 7.98 (1H, d, ^3^*J*_HH_ 7.2 Hz, H-8), 7.15 (1H, d, ^3^*J*_HH_ 7.3 Hz, H-3), 7.03–6.98 (2H, m, H-18), 6.93 (1H, d, ^3^*J*_HH_ 7.2 Hz, H-3), 6.96–6.87 (3H, m, H-17,19), 3.47 (2H, br s, H-13,13′), 3.09–3.01 (4H, m, H-11,12), 1.34 (6H, dd, ^3^*J*_HP_ 17.1, ^3^*J*_HH_ 6.9 Hz, H-14,14′), 1.04 (6H, dd, ^3^*J*_HP_ 17.7, ^3^*J*_HH_ 6.9 Hz, H-15/15′). **^13^C DEPTQ NMR** (125.8 MHz, C_6_D_5_Br, 368 K) δ_C_ 151.4 (d, ^4^*J*_CP_ 2.5 Hz, qC-4), 148.2 (s, qC-6), 142.2 (s, C-8), 141.0 (d, ^3^*J*_CP_ 8.6 Hz, qC-5), 135.8 (s, qC-10), 130.0 (s, C-18), 128.8 (s, C-17), 125.9 (s, C-19), 124.1 (d, ^1^*J*_CP_ 64.2 Hz, C-1), 120.8 (s, C-7), 118.3 (d, ^3^*J*_CP_ 12.3 Hz, C-3), 30.1 (d, ^1^*J*_CP_ 50.2 Hz, C-13,13′), 29.8 (s, C-11/12), 29.8 (s, C-11/12), 17.8 (s, C-14,14′), 17.5 (s, C-15/15′). **^31^P{^1^H} NMR** (202.4 MHz, CDCl_3_, 253 K) δ_P_ 82.6 (s), 64.7 (s). **^31^P{^1^H} NMR** (202.4 MHz, CDCl_3_, 295 K) δ_P_ 82.8 (s), 65.1 (s). **^31^P{^1^H} NMR** (202.4 MHz, C_6_D_5_Br, 363 K) δ_P_ 71.9 (s). **^77^Se{^1^H} NMR** (95.4 MHz, CDCl_3_, 253 K) δ_Se_ 418.9 (s), 413.2 (s). **^77^Se{^1^H} NMR** (95.4 MHz, CDCl_3_, 293 K) δ_Se_ 422.9 (br s), 418.2 (br s). **^77^Se{^1^H} NMR** (95.4 MHz, C_6_D_5_Br, 368 K) δ_Se_ 433.2 (s). **IR**: ν_max_ ATR/cm^−1^ 3055w (ν_CH_), 2958w (ν_CH_), 1578m (ν_C=C_), 1477m (ν_C=C_), 1022m, 744s, 687vs (ν_P=S_). **HRMS** (APCI+): *m*/*z* (%) Cacld. for C_24_H_28_PSSe: 459.0815, found: 459.0814 (100) [M+H].

#### 4.2.4. Synthesis of **1-Se**

A suspension of **1_Ph_** (1.50 g, 3.52 mmol) and selenium (276 mg, 3.50 mmol) in toluene (30 mL) was heated under reflux for fifteen hours. The solution was cooled to ambient conditions and all volatiles removed in vacuo to afford the crude product. Recrystallization from dichloromethane:*n*-hexane (1:3 *v*/*v*) at −20 °C afforded yellow analytically pure crystals of **1-Se** (1.10 g, 62%) (melting with decomp. 237–242 °C). These crystals were suitable for single-crystal X-ray diffraction. **Elemental Analysis**: Cacld. (%) for C_24_H_27_PSe_2_: C 57.15, H 5.40, found: C 56.93, H 5.36. **^1^H NMR** (500.1 MHz, C_6_D_5_Br, 363 K) δ_H_ 8.02 (1H, d, ^3^*J*_HH_ 7.2 Hz, H-8), 7.12 (1H, d, ^3^*J*_HH_ 7.4 Hz, H-3), 7.00–6.92 (3H, m, H-7,18), 6.92–6.85 (3H, m, H-17,19), 3.55 (2H, br s, H-13,13′), 3.16–2.90 (4H, m, H-11,12), 1.33 (6H, dd, ^3^*J*_HP_ 17.5, ^3^*J*_HH_ 6.8 Hz, H-14,14′), 1.04 (6H, dd, ^3^*J*_HP_ 18.3, ^3^*J*_HH_ 6.9 Hz, H-15,15′). **^13^C DEPTQ NMR** (126.8 MHz, C_6_D_5_Br, 363 K) δ_C_ 151.6 (d, ^4^*J*_CP_ 2.6 Hz, qC-4), 148.3* (s, qC-6), 142.4 (s, C-8), 141.0 (d, ^3^*J*_CP_ 8.2 Hz, qC-5), 135.8 (s, qC-10), 129.8 (s, C-18), 128.8 (s, C-17), 125.9 (s, C-18*), 123.4 (s, qC-9), 121.9* (s, qC-1), 120.9 (s, C-7), 118.3 (d, ^3^*J*_CP_ 12.4 Hz, C-3), 29.8 (s, C-11/12), 29.6 (s, C-11/12), 29.3 (d, ^1^*J*_CP_ 42.5 Hz, C-13,13′), 18.8* (s, C-14,14′), 18.7* (s, C-15,15′). **^31^P{^1^H} NMR** (202.5 MHz, CDCl_3_, 253 K), δ_P_ 86.2 (s, ^1^*J*_PSe_ 682.9 Hz), 58.0 (s, ^1^*J*_PSe_ 681.3 Hz). **^31^P{^1^H} NMR** (202.5 MHz, CDCl_3_, 295 K) δ_P_ 86.3 (br s), 58.4 (br s). **^31^P{^1^H} NMR** (202.5 MHz, C_6_D_5_Br, 363 K), δ_P_ 68.3 (br s). **^31^P{^1^H} SS-MAS NMR** (162.0 MHz) 60.2 (s, ^1^*J*_PSe_ 699.2 Hz). **^77^Se{^1^H} NMR** (95.4 MHz, CDCl_3_, 255 K) δ_Se_ 422.8 (s), 415.3 (s, ^5TS^*J*_SeSe_ 182.0 Hz), −362.6 (d, ^1^*J*_SeP_ 681.5 Hz), −452.4 (d, ^1^*J*_SeP_ 683.6 Hz). **^77^Se{^1^H} NMR** (95.4 MHz, CDCl_3_, 293 K) δ_Se_ 426.2 (br s), 419.0 (br s), −358.5 (d, ^1^*J*_SeP_ 696.4 Hz), −451.0 (d, ^1^*J*_SeP_ 693.4 Hz). **^77^Se{^1^H} NMR** (95.4 MHz, C_6_D_5_Br, 363 K) δ_Se_ 433.0 (s). **^77^Se{^1^H} SS-MAS NMR** (76.3 MHz) 431.6 (s), −353.9 (d, ^1^*J*_PSe_ 700.8 Hz). **IR**: ν_max_ ATR/cm^−1^ 3047w (ν_CH_), 2962w (ν_CH_), 1601m (ν_C=C_), 1473m (ν_C=C_), 1018m, 744s, 636s. **HRMS** (APCI+): *m*/*z* (%) Cacld. for C_24_H_28_PSe_2_: 507.0263, found: 507.0264 (100) [M+H]. Note: ^13^C signals denotated with * were observed in the 2D ^1^H–^13^C HMBC only.

### 4.3. Crystallographic Details

The crystallographic data for **1-O** were collected using a Rigaku XtaLAB P200 diffractometer using multi-layer mirror monochromated Mo Kα radiation at −180 °C (±1). The crystallographic data for **1-S** were collected using a Rigaku XtaLAB P100 diffractometer using multi-layer mirror monochromated Cu Kα radiation at −100 °C (±1). The crystallographic data for **1-O2** and **1-Se** were collected using a Rigaku SCX mini diffractometer using graphite monochromated Mo Kα radiation at −100 °C (±1) (Mo Kα = λ = 0.71073 Å; Cu Kα = λ = 1.54184 Å).

Intensity data were collected using ω steps accumulating area detector frames spanning at least a hemisphere of reciprocal space. All data were corrected for Lorentz, polarization, and long-term intensity fluctuations. Absorption effects were corrected on the basis of multiple equivalent reflections. The structures were solved by direct methods [26]. Non-hydrogen atoms were refined anisotropically, and hydrogen atoms were refined using the riding model.

The crystal structures were refined by full-matrix least squares against F2 (SHELXL) [27,28] using the CrystalStructure GUI [29]. Searches of the Cambridge Structural Database (CSD) were performed using the webCSD [30]. Images and manipulations of crystal structures and computed rotamers were obtained using OLEX-2 [31].

### 4.4. Computational Details

Geometries were fully optimized at the B3LYP level [32,33] (using a fine integration grid, i.e., 75 radial shells with 302 angular points per shell) with Curtis and Binning’s 962(d) basis [34] on Se and 6-31+G(d,p) elsewhere. The solid-state structure was used as starting point for the optimizations of conformer **1-Se(A)**. The nature of the stationary points was verified by computation of the harmonic frequencies at the same level of theory, which were also used to compute thermodynamic corrections to obtain enthalpies and free energies (standard pressure and temperature). The structures were then re-optimized at the dispersion-corrected B3LYP-D3 [35] level using the same basis set and Becke–Johnson damping [36,37]. Single-point energies were refined for the B3LYP-D3 structures at the B3LYP-D3 level using 962+(d,f) basis on Se, i.e., including the recommended [38] diffuse s and p set and the f-function, and 6-311+G(d,p) elsewhere; an implicit solvent model was used in these single-point calculations, namely the Conductor-Like Polarizable Continuum Model (CPCM) [39,40], using the default settings in Gaussian09 and the parameters of bromobenzene. Wiberg bond indices (WBIs) were computed at that level from natural bond orbital (NBO) analysis. The WBI is a measure for the covalent character of a bond and adopts values close to 1 and 2 for true single and double bonds, respectively [41]. This and similar levels have performed well in previous studies of related acenaphthene chalcogen and pnictogen compounds [2,3,4,5,6,7,13,15,17]. Magnetic shieldings and spin–spin coupling constants (SSCCs) were computed at the GIAO-B3LYP level using IGLO DZ basis on H atoms and IGLO-basis II everywhere else (denoted ILGO-II), which was designed for computation of magnetic properties [42] and the CPCM model with the parameters of chloroform. The relative ^77^Se shifts were referenced relative to Me_2_Se (computed σ = 1652.1 ppm at the same level). Because the experimental standard for ^31^P NMR, concentrated phosphoric acid, is difficult to model computationally, chemical shifts were first referenced to Ph_3_PSe (computed σ = 235.1 ppm) and converted to the usual δ scale using the experimental chemical shift of that compound in CDCl_3_, 43.2 ppm [43]. In the computations of SSCCs, the basis set was uncontracted for evaluating the Fermi contact contribution (keyword NMR = (Spin–Spin, Mixed) in Gaussian). All computations were performed using the Gaussian09 suite of programs [44].

## 5. Conclusions

A series of phosphorus and selenium *peri*-substituted acenaphthenes with the phosphorus atom oxidized by oxygen, sulfur, and selenium was synthesized and characterized by single-crystal X-ray diffraction and multinuclear NMR spectroscopy. For the Se(II) species, there were two major rotational conformers in solution, as identified by Variable-Temperature NMR experiments and supported with DFT calculations. Only one of these conformations was present in the solid state, as verified by X-ray crystallography and solid-state NMR spectroscopy.

## Figures and Tables

**Table 1:** Selected bond lengths (ångströms (Å)) and angles (degrees, °) for **1-O**, **1-S**, **1-Se**, and **1-O2**.

Compound	1-O	1-S	1-Se	1-O2 ^[b]^
*peri*-region bond distances				
P1···Se1	3.322(2)	3.4863(5)	3.5012(7)	3.578(1) [3.610(1)]
P1-E	1.491(6)	1.9657(5)	2.1219(7)	1.487(4) [1.492(4)]
Se1···E	2.825(6)	3.2272(5)	3.2829(6)	2.646(3) [2.625(3)]
Se1-O1	−	−	−	1.669(3) [1.671(4)]
*peri*-region bond angles				
C9-P1-E	110.7(4)	112.39(5)	112.40(8)	112.7(2) [113.1(2)]
P1-E-Se1	95.7(3)	80.36(2)	77.47(2)	117.1(2) [118.5(2)]
E-Se1-C19	165.4(3)	164.53(5)	166.29(8)	84.6(2) [86.4(2)]
O2-Se1-O1	−	−	−	169.3(1) [169.2(2)]
C1-Se1-C19	99.3(3)	97.01(6)	96.8(1)	97.7(2) [95.4(2)]
Splay ^[a]^	18.5	19.9	20.2	28.1 [29.1]
dihedral angles				
C9-C10-C5-C4	174.8(8)	173.7(1)	173.2(2)	178.3(5) [177.4(5)]
P1-C9···C1-Se1	22.2(5)	31.87(8)	32.0(1)	1.8(3) [1.7(3)]
out-of-plane displacements				
P1	0.508	0.605	0.593	0.065 [0.007]
Se1	−0.393	−0.700	−0.725	0.006 [0.128]

^[a]^ splay angle = sum of the bay region angles—360. ^[b]^ values in square parentheses are for the 2nd molecule in the asymmetric unit.

**Table 2:** Computed ^a^ relative energies ΔE, enthalpies ΔH, and free energies Δ*G*^a^ for selected rotamers in kJ mol^−1^ relative to **1-Se(A)**, as well as computed ^a^ and experimental ^b^ (in italics) δ(^31^P) and δ(^77^Se) chemical shifts of **1-Se(A)** and **1-Se(D)**.

Molecule	Δ*E*	Δ*H*^298^	Δ*G*^298^	δ(P)	δ(Se = P)	δ(SePh)
**1-Se(A)**	0	0	0	61.7 *58.0* ^b^	−389.9 *−362.6* ^b^	385.1 *415.3* ^b^
**1-Se(B)**	19.0	19.7	23.5			
**1-Se(C)**	32.8	33.8	34.3			
**1-Se(D)**	−2.6	−2.4	−2.4	89.1 *86.2* ^b^	−555.7 *−452.4* ^b^	433.8 *422.8* ^b^

^a^ Energies at B3LYP-D3/6-311+G(d,p)/CPCM(C_6_H_5_Br)//B3LYP-D3/6-31+G(d,p) level, thermodynamic corrections from B3LYP/6-31+G(d,p); chemical shifts at GIAO-B3LYP/ILGO-II`/CPM(CHCl_3_)//B3LYP-D3/6-31+G(d,p) level ^b^ 235 K in CDCl_3_ (this work).

## Data Availability

Accession codes CCDC 2298921-2298924 contain the supplementary crystallographic data for this paper. These data can be obtained free of charge via www.ccdc.cam.ac.uk/data_request/cif or by emailing data_request@ccdc.cam.ac.uk. The research data underpinning this publication can be accessed at https://doi.org/10.17630/8fe507af-e08a-4ce8-8edb-426149d527e6.

## Supplementary Materials

The following supporting information can be downloaded at: https://www.mdpi.com/article/10.3390/molecules28217297/s1, Figures S1–S5: Additional NMR spectra; Figures S6–S9: IR spectra of compounds; Table S1: Crystal and structure refinement data; computational detail: Cartesian coordinates in Å, B3LYP/6-31+G(d,p) optimized for rotamers of **1-Se**.

## References

[1] Chalmers B.A., Athukorala Arachcige K.S., Prentis J.K.D., Knight F.R., Kilian P., Slawin A.M.Z., Woollins J.D. (2014). Sterically Encumbered Tin and Phosphorus *peri*-Substituted Acenaphthenes. Inorg. Chem..

[2] Chalmers B.A., Nejman P.S., Llewellyn A.V., Felaar A.M., Griffiths B.L., Portman E.I., Gordon E.-J.L., Fan K.J.H., Woollins J.D., Bühl M. (2018). A Study of Through-Space and Through-Bond J_PP_ Coupling in a Rigid Nonsymmetrical Bis(phosphine) and Its Metal Complexes. Inorg. Chem..

[3] Knight F.R., Randall R.A.M., Roemmele T.L., Boeré R.T., Bode B.E., Crawford L., Bühl M., Slawin A.M.Z., Woollins J.D. (2013). Electrochemically Informed Synthesis: Oxidation versus Coordination of 5,6-Bis(phenylchalcogeno)acenaphthenes. ChemPhysChem.

[4] Chalmers B.A., Bühl M., Athukorala Arachcige K.S., Slawin A.M.Z., Kilian P. (2015). A Strutural, Spectroscopic, and Computational Examination of the Dative Interaction in Constrained Phosphine-Stibines and Phosphine-Stiboranes. Chem. Eur. J..

[5] Chalmers B.A., Bühl M., Athukorala Arachcige K.S., Slawin A.M.Z., Kilian P. (2014). Geometrically Enforced Donor-Facilitated De-hydrocoupling Leading to an Isolable Arsanylidine-Phosphorane. J. Am. Chem. Soc..

[6] Nejman P.S., Curzon T.E., Bühl M., McKay D., Woollins J.D., Ashbrook S.E., Cordes D.B., Slawin A.M.Z., Kilian P. (2020). Phosphorus-Bismuth *peri*-Substituted Acenaphthenes: A Synthetic, Structural, and Computational Study. Inorg. Chem..

[7] Nordheider A., Hupf E., Chalmers B.A., Knight F.R., Bühl M., Mebs S., Checinska L., Lork E., Camacho P.S., Ashbrook S.E. (2015). *Peri*-Substituted Phosphorus–Tellurium Systems—An Experimental and Theoretical Investigation of the P···Te through-Space Interaction. Inorg. Chem..

[8] Hupf E., Lork E., Mebs S., Checinska L., Beckmann J. (2014). Probing Donor−Acceptor Interactions in *peri*-Substituted Diphenylphosphinoacenaphthyl−Element Dichlorides of Group 13 and 15 elements. Organometallics.

[9] Hupf E., Lork E., Mebs S., Beckmann J. (2015). 6-Diphenylphosphinoacenaphth-5-yl-mercurials as Ligands for d10 Metals. Observation of Closed-Shell Interactions of the Type Hg(II)···M; M = Hg(II), Ag(I), Au(I). Inorg. Chem..

[10] Furan S., Vogt M., Winkels K., Lork E., Mebs S., Hupf E., Beckmann J. (2021). (6-Diphenylphosphinoacenaphth-5-yl)indium and -nickel Compounds: Synthesis, Structure, Transmetalation, and Cross-Coupling Reactions. Organometallics.

[11] Kordts N., Künzler S., Rathjen S., Sieling T., Großekappenberg H., Schmidtmann M., Müller T. (2017). Silyl Chalconium Ions: Synthesis, Structure and Application in Hydrodefluorination Reactions. Chem. Eur. J..

[12] Hierso J.C. (2014). Indirect Nonbonded Nuclear Spin−Spin Coupling: A Guide for the Recognition and Understanding of “Through-Space” NMR J Constants in Small Organic, Organometallics, and Coordination Compounds. Chem. Rev..

[13] Athukorala Arachcige K.S., Camacho P.S., Ray M.J., Chalmers B.A., Knight F.R., Ashbrook S.E., Bühl M., Kilian P., Slawin A.M.Z., Woollins J.D. (2014). Sterically Restricted Tin Phosphines, Stabilised by Weak Intramolecular Donor-Acceptor Interactions. Organometallics.

[14] Hupf E., Lork E., Mebs S., Beckmann J. (2014). Intramolecularly Coordinated (6-(Diphenylphosphino)acenapth-5-yl)stannanes. Repulsion vs Attraction of P- and Sn-Containing Substituents in the *peri* Positions. Organometallics.

[15] Knight F.R., Fuller A.L., Bühl M., Slawin A.M.Z., Woollins J.D. (2010). Sterically Crowded *peri*-Substituted Naphthalene Phosphines and their P^V^ Derivatives. Chem. Eur. J..

[16] Malkina O.L., Hierso J.-C., Malkin V.G. (2022). Distinguishing “Through-Space” from “Through-Bonds” Contribution in Indirect Nuclear Spin−Spin Coupling; General Approaches Applied to Complex *J*_PP_ and *J*_PSe_ Scalar Couplings. J. Am. Chem. Soc..

[17] Zhang L., Christie F.A., Tarcza A.E., Lancaster H.G., Taylor L.J., Bühl M., Malkinia O.L., Woollins J.D., Carpenter-Warren C.L., Cordes D.B. (2023). Phosphine and Selenoether *peri*-Substituted Acenaphthenes and Their Transition-Metal Complexes: Structural and NMR Investigations. Inorg. Chem..

[18] Chernick C.L., Skinner H.A. (1956). 285. Thermochemistry of organophosphorus compounds. Part II. Triethyl phoshate, tripropylphosphine oxide, and tributylphosphine oxide. J. Chem. Soc..

[19] Bartlett P.D., Meguerian G. (1956). Reactions of Elemental Sulfur. I. The Uncatalysed Teaction of Sulfur with Triarylphosphines. J. Am. Chem. Soc..

[20] Capps K.B., Wixmerten B., Bauer A., Hoff C.D. (1998). Thermochemistry of Sulfur Atom Transfer. Enthalpies of Reaction of Phosphines with Sulfur, Selenium, and Tellurium, and of Desulfurization of Triphenylarsenic Sulfide, Triphenylantimony Sulfide, and Benzyl Trisulfide. Inorg. Chem..

[21] Cinderalla A.P., Vulovic B., Watson D.A. (2017). Palladium-Catalysed Cross-Coupling of Silyl Electrophiles with Akylzinc Halides: A Silyl-Negishi Reaction. J. Am. Chem. Soc..

[22] Kilian P., Milton H.L., Slawin A.M.Z., Woollins J.D. (2004). Chlorides, Oxochlorides, and Oxoacids of 1,8-Diphosphanaphthalene: A System with Imposed Close P···P Interaction. Inorg. Chem..

[23] Eventova V.A., Belov K.V., Efimov S.V., Khodov I.A. (2023). Conformational Screening of Arbidol Solvates: Investigation via 2D NOESY. Pharmaceutics.

[24] Pyykko P., Atsumi M. (2009). Molecular Single-Bond Covalent Radii for Elements 1–118. Chem. Eur. J..

[25] Mantina M., Chamberlin A.C., Valero R., Cramer C.J., Truhlar D.G. (2009). Consistent ver der Waals Radii for the Whole Main Group. J. Phys. Chem. A.

[26] Burla M.C., Caliandro R., Camalli M., Carrozzini B., Cascarano G.L., Giacovazzo C., Mallamo M., Mazzone A., Polidori G., Spagna R. (2012). SIR2011: A new package for crystal structure determination and refinement. J. Appl. Crystallogr..

[27] Sheldrick G.M. (2015). Crystal structure refinement with *SHELXL*. Acta Crystallogr. C.

[28] Sheldrick G.M. (2015). SHELXT—Integrated space-group and crystal-structure determination. Acta Crystallogr. A.

[29] (2018). CrystalStructure 4.3.0.

[30] Groom C.R., Bruno I.J., Lightfoot M.P., Ward S.C. (2016). The Cambridge Structural Database. Acta Crystallogr. B.

[31] Dolomanov O.V., Bourhis L.J., Gildea R.J., Howard J.A.K., Puschmann H. (2009). OLEX2: A complete structure solution, refinement and analysis program. J. Appl. Crystallogr..

[32] Becke A.D. (1993). Density-functional thermochemistry. III. The role of exact exchange. J. Chem. Phys..

[33] Lee C., Yang W., Parr R.G. (1988). Development of the Colle-Salvetti correlation-energy formula into a function of the electron density. Phys. Rev. B.

[34] Binning R.C., Curtiss L.A. (1990). Compact contracted basis sets for third-row atoms: Ga–Kr. J. Comput. Chem..

[35] Grimme S., Antony J., Ehrlich S., Kreig H. (2010). A consistent and accurate ab initio parametrization of density function dispersion correction (DFT-D) for the 94 elements H-Pu. J. Chem. Phys..

[36] Becke A.D., Johnson E.R. (2005). Exchange-hole dipole moment and the dispersion interaction. J. Chem. Phys..

[37] Johnson E.R., Becke A.D. (2006). A post-Hartree-Fock model of intermolecular interactions: Inclusion of higher-order corrections. J. Chem. Phys..

[38] Reed A.E., Curtiss L.A., Weinhold F. (1988). Intermolecular Interactions from a Natural Bond Orbital, Donor-Acceptor Viewpoint. Chem. Rev..

[39] Barone V., Cossi M. (1998). Quantum Calculation of Molecular Energies and Energy Gradients in Solution by a Conductor Solvent Model. J. Phys. Chem. A.

[40] Cossi M., Rega N., Scalmani G., Barone V. (2003). Energies, structures, and electronic properties of molecules in solution with the C-PCM solvation model. J. Comput. Chem..

[41] Wiberg K.B. (1968). Application of the pople-santry-segal CNDO method to the cyclopropylcarbinyl and cyclobutyl cation and to bicyclobutane. Tetrahedron.

[42] Kutzelnigg W., Fleischer U., Schindler M. (1990). The IGLO-Method: Ab Initio Calculation and Interpretation of NMR Chemical Shifts and Magnetic Susceptibilities. NMR Basic Principles and Progress.

[43] Albright T.A., Freeman W.J., Schweizer E.E. (1975). Nuclear Magnetic Resonance Studies. IV. The Carbon and Phosphorus Nuclear Magnetic Resonance of Phosphine Oxides and Related Compounds. J. Org. Chem..

[44] Frisch M.J., Trucks G.W., Schlegel H.B., Scuseria G.E., Robb M.A., Cheeseman J.R., Scalmani G., Barone V., Mennucci B., Petersson G.A. (2009). Gaussian 09.

