# Supplementary material for: Constrained Phosphine Chalcogenide Selenoethers Supported by peri-Substitution
Source: Molecules. 2023 Oct 27;28(21):7297. doi: 10.3390/molecules28217297 (PMC10650533; doi:10.3390/molecules28217297)

checkCIF/PLATON report

Structure factors have been supplied for datablock(s) 1-O.

THIS REPORT IS FOR GUIDANCE ONLY. IF USED AS PART OF A REVIEW PROCEDURE FOR PUBLICATION, IT SHOULD NOT REPLACE THE EXPERTISE OF AN EXPERIENCED CRYSTALLOGRAPHIC REFEREE.

No syntax errors found. CIF dictionary Interpreting this report

Datablock: 1-O.

Bond precision: C-C = 0.0122 A Wavelength=0.71075

Cell:	a=8.886(2)	b=11.950(2)	c=19.754(4)
	alpha=90	beta=96.223(6)	gamma=90
Temperature:	93 K		

	Calculated	Reported
Volume	2085.3(7)	2085.3(7)
Space group	P 21/n	P 1 21/n 1
Hall group	-P 2yn	-P 2yn
Moiety formula	C24 H27 O P Se	C24 H27 O P Se
Sum formula	C24 H27 O P Se	C24 H27 O P Se
Mr	441.39	441.41
Dx,g cm-3	1.406	1.406
Z	4	4
Mu (mm-1)	1.889	1.889
F000	912.0	912.0
F000'	912.31	
h,k,lmax	10,14,23	10,14,23
Nref	3836	3760
Tmin,Tmax	0.692,0.685	0.457,0.685
Tmin'	0.679	

Correction method= # Reported T Limits: Tmin=0.457 Tmax=0.685
AbsCorr = MULTI-SCAN

Data completeness= 0.980 Theta(max)= 25.400

R(reflections)= 0.0713(3526) wR2(reflections)= 0.2117(3760)

S = 1.252 Npar= 248

The following ALERTS were generated. Each ALERT has the format
test-name_ALERT_alert-type_alert-level.
Click on the hyperlinks for more details of the test.

Alert level C

```
PLAT094_ALERT_2_C Ratio of Maximum / Minimum Residual Density ....       3.06 Report
PLAT341_ALERT_3_C Low Bond Precision on  C-C Bonds ..............    0.01221 Ang.
PLAT906_ALERT_3_C Large K Value in the Analysis of Variance ......      7.940 Check
PLAT906_ALERT_3_C Large K Value in the Analysis of Variance ......      2.389 Check
PLAT911_ALERT_3_C Missing FCF Refl Between Thmin & STh/L=    0.600         68 Report
PLAT918_ALERT_3_C Reflection(s) with I(obs) much Smaller I(calc) .          1 Check
PLAT934_ALERT_3_C Number of (Iobs-Icalc)/Sigma(W) > 10 Outliers ..          1 Check
PLAT971_ALERT_2_C Check Calcd Resid. Dens.  1.48Ang From C18             2.43 eA-3
PLAT977_ALERT_2_C Check Negative Difference Density on H18B      .      -0.34 eA-3
PLAT977_ALERT_2_C Check Negative Difference Density on H22       .      -0.36 eA-3
```

Alert level G

```
PLAT083_ALERT_2_G SHELXL Second Parameter in WGHT  Unusually Large      44.08 Why ?
PLAT912_ALERT_4_G Missing # of FCF Reflections Above STh/L=  0.600          9 Note
PLAT930_ALERT_2_G FCF-based   Twin Law         [ 1 0 4] Est.d BASF       0.13 Check
PLAT931_ALERT_5_G CIFcalcFCF  Twin Law         [ 1 0 4] Est.d BASF       0.13 Check
PLAT933_ALERT_2_G Number of HKL-OMIT Records in Embedded .res File          2 Note
PLAT978_ALERT_2_G Number C-C Bonds with Positive Residual Density.          1 Info
```

0 **ALERT level A** = Most likely a serious problem - resolve or explain
0 **ALERT level B** = A potentially serious problem, consider carefully
10 **ALERT level C** = Check. Ensure it is not caused by an omission or oversight
6 **ALERT level G** = General information/check it is not something unexpected

0 ALERT type 1 CIF construction/syntax error, inconsistent or missing data
8 ALERT type 2 Indicator that the structure model may be wrong or deficient
6 ALERT type 3 Indicator that the structure quality may be low
1 ALERT type 4 Improvement, methodology, query or suggestion
1 ALERT type 5 Informative message, check

It is advisable to attempt to resolve as many as possible of the alerts in all categories. Often the minor alerts point to easily fixed oversights, errors and omissions in your CIF or refinement strategy, so attention to these fine details can be worthwhile. In order to resolve some of the more serious problems it may be necessary to carry out additional measurements or structure refinements. However, the purpose of your study may justify the reported deviations and the more serious of these should normally be commented upon in the discussion or experimental section of a paper or in the "special_details" fields of the CIF. checkCIF was carefully designed to identify outliers and unusual parameters, but every test has its limitations and alerts that are not important in a particular case may appear. Conversely, the absence of alerts does not guarantee there are no aspects of the results needing attention. It is up to the individual to critically assess their own results and, if necessary, seek expert advice.

Publication of your CIF in IUCr journals

A basic structural check has been run on your CIF. These basic checks will be run on all CIFs submitted for publication in IUCr journals (*Acta Crystallographica*, *Journal of Applied Crystallography*, *Journal of Synchrotron Radiation*); however, if you intend to submit to *Acta Crystallographica Section C* or *E* or *IUCrData*, you should make sure that full publication checks are run on the final version of your CIF prior to submission.

Publication of your CIF in other journals

Please refer to the *Notes for Authors* of the relevant journal for any special instructions relating to CIF submission.

PLATON version of 10/05/2023; check.def file version of 10/05/2023

Datablock 1-O. - ellipsoid plot

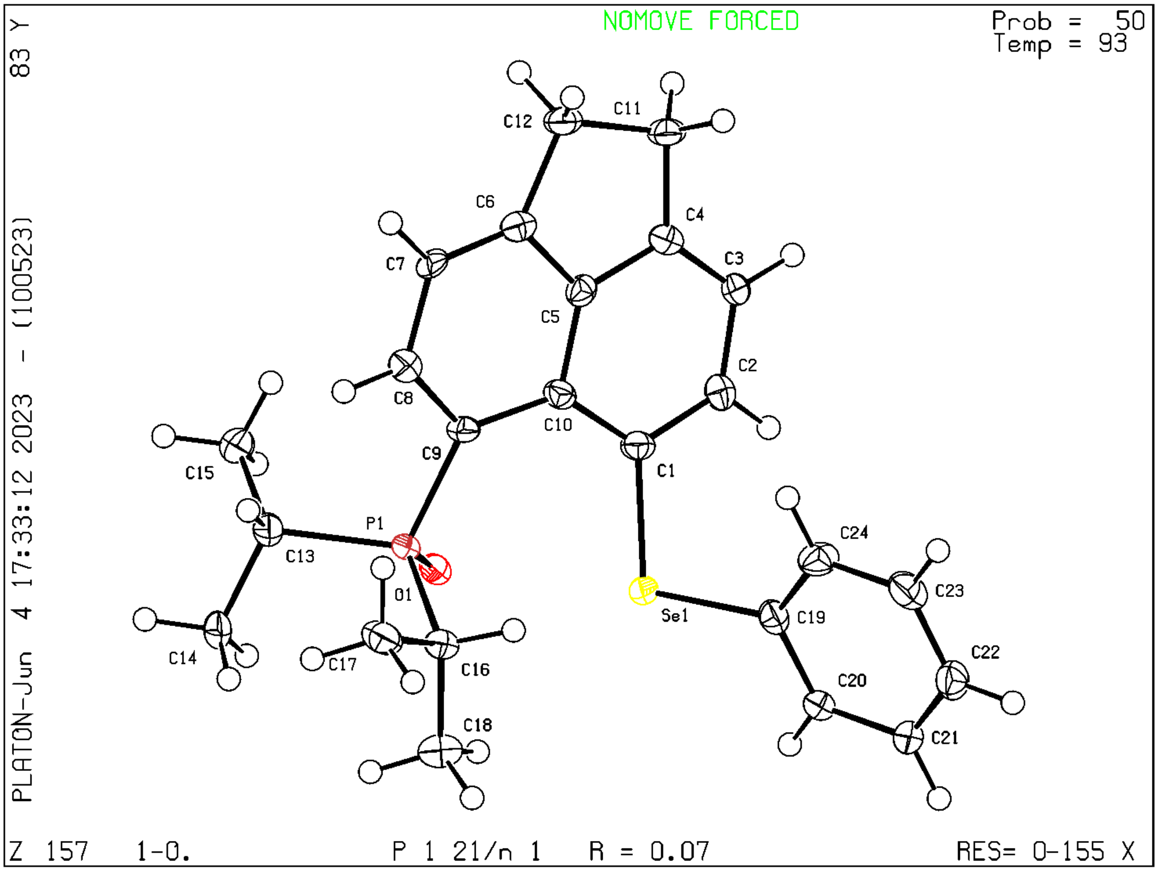

Supplement: Supplementary file 1 [file molecules-28-07297-s001.zip › Supporting Information/SC XRD/1-Ocheckcif.pdf]
