# Supplementary material for: Constrained Phosphine Chalcogenide Selenoethers Supported by peri-Substitution
Source: Molecules. 2023 Oct 27;28(21):7297. doi: 10.3390/molecules28217297 (PMC10650533; doi:10.3390/molecules28217297)
Supplement: Supplementary file 1 [file molecules-28-07297-s001.zip › Supporting Information/Supporting Information v5.pdf]

Supporting Information for:

Constrained Phosphine Chalcogenide Selenoethers Supported by *peri*-Substitution

Anna E. Tarcza, Alexandra M. Z. Slawin, Cameron L. Carpenter-Warren, Michael Bühl, Petr Kilian, Brian A. Chalmers*

EaStCHEM School of Chemistry, University of St Andrews, North Haugh, St Andrews, Fife KY16 9ST, UK

*Correspondence: bac8@st-andrews.ac.uk; Tel.: +44-1334-463785

Additional NMR Spectra

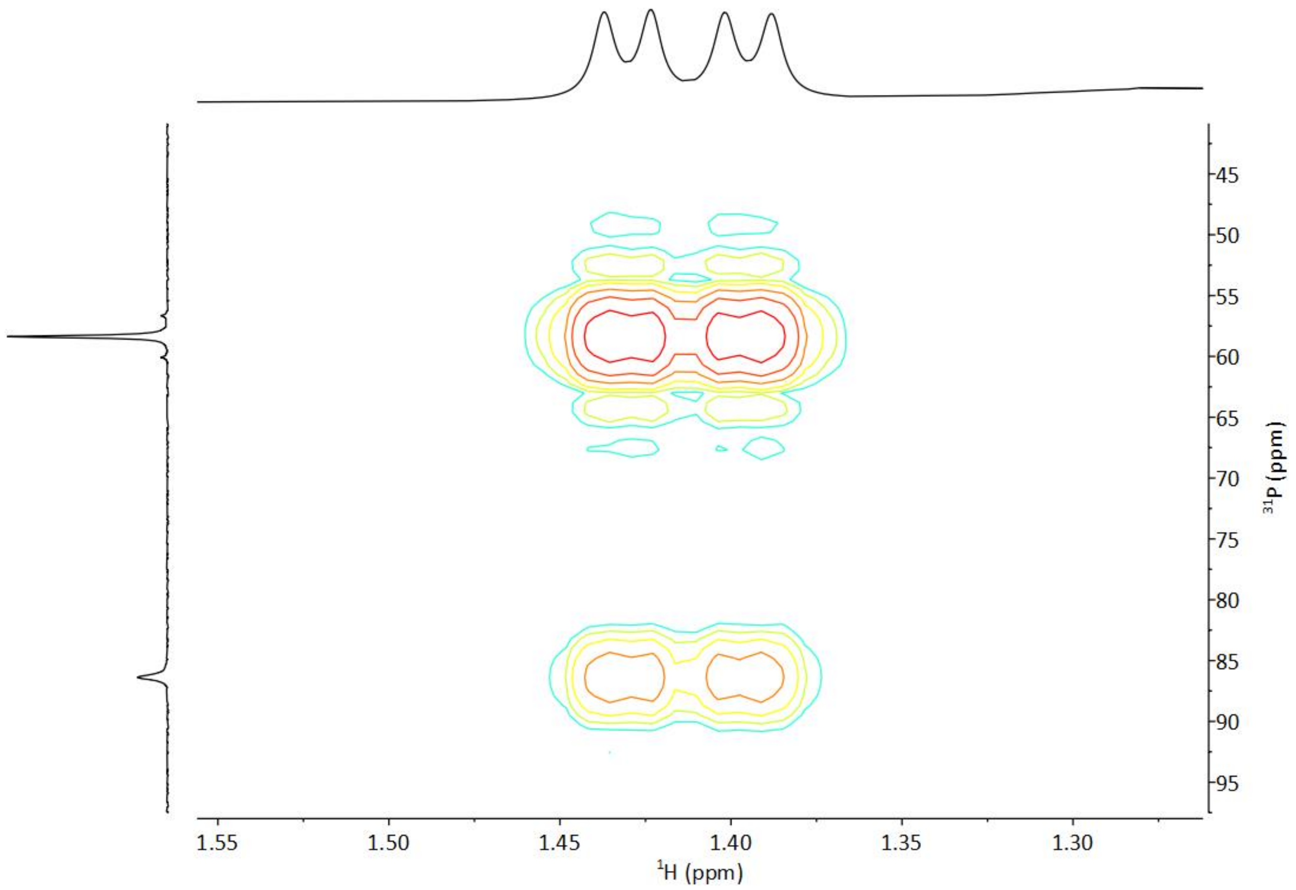

Figure S1. ^{1}H–^{31}P HMBC NMR spectrum of **1-Se**.

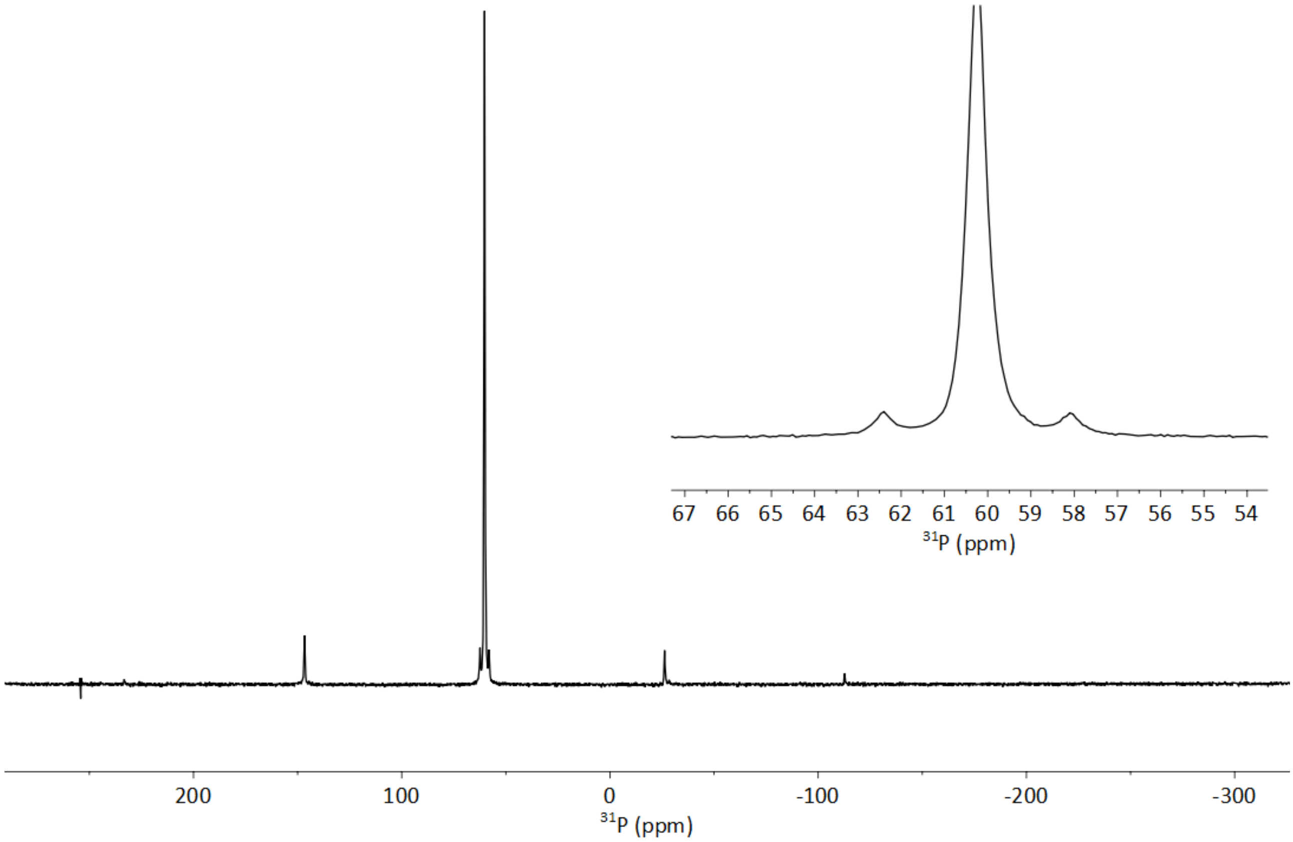

Figure S2. The $^{31}P\{^{1}H\}$ SS-MAS NMR spectrum of 1-Se recorded at 162.0 MHz with expansion of the isotropic peak showing the ^{77}Se satellites.

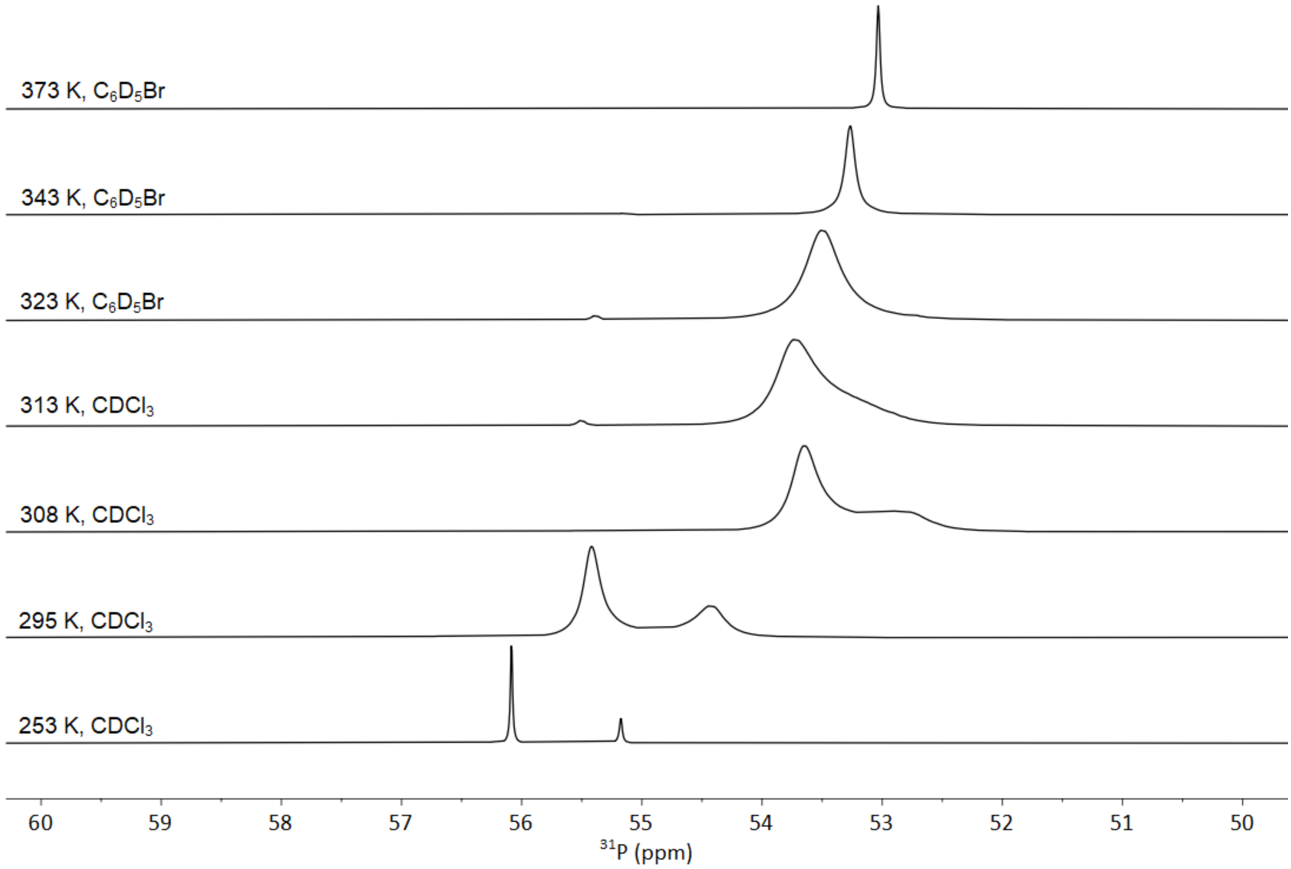

Figure S3. The $^{31}P\{^{1}H\}$ VT NMR spectra of **1-O** with solvent and temperatures indicated (acquired at 202.5 MHz).

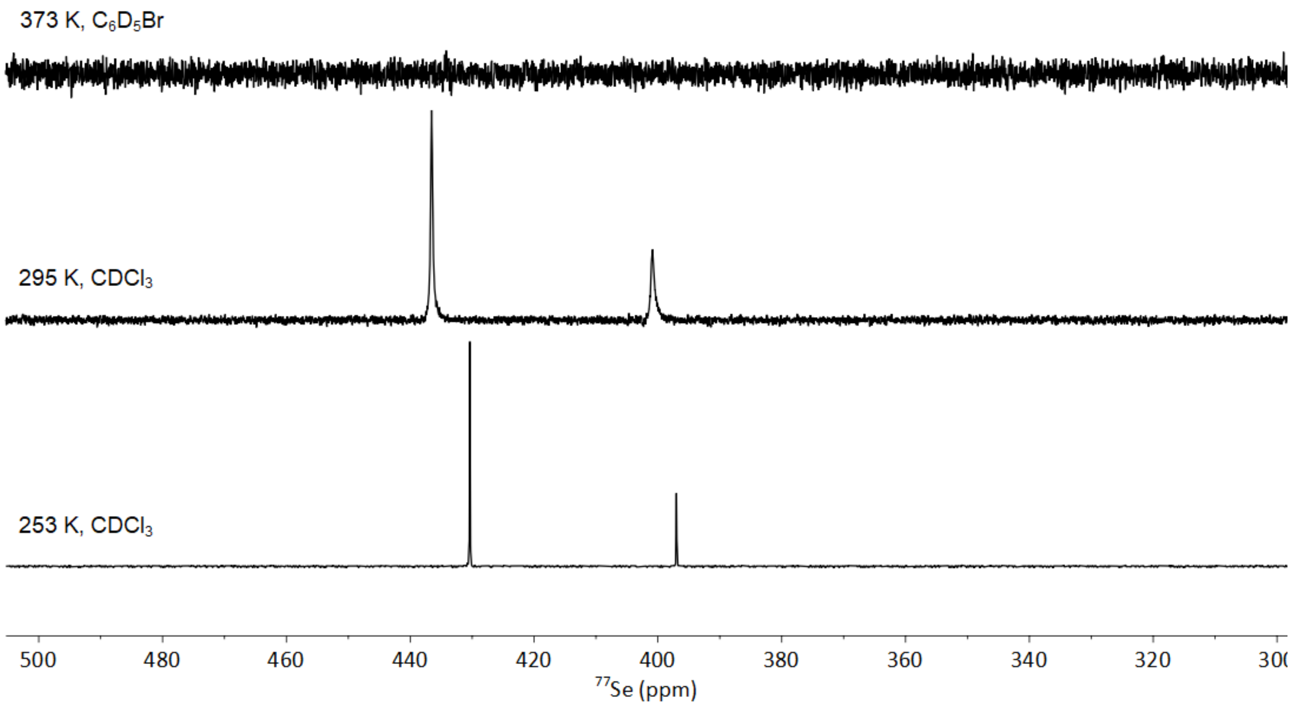

Figure S4. The ^{77}Se{^{1}H} VT NMR spectra of **1-Se** with solvent and temperatures indicated (acquired at 95.4 MHz)

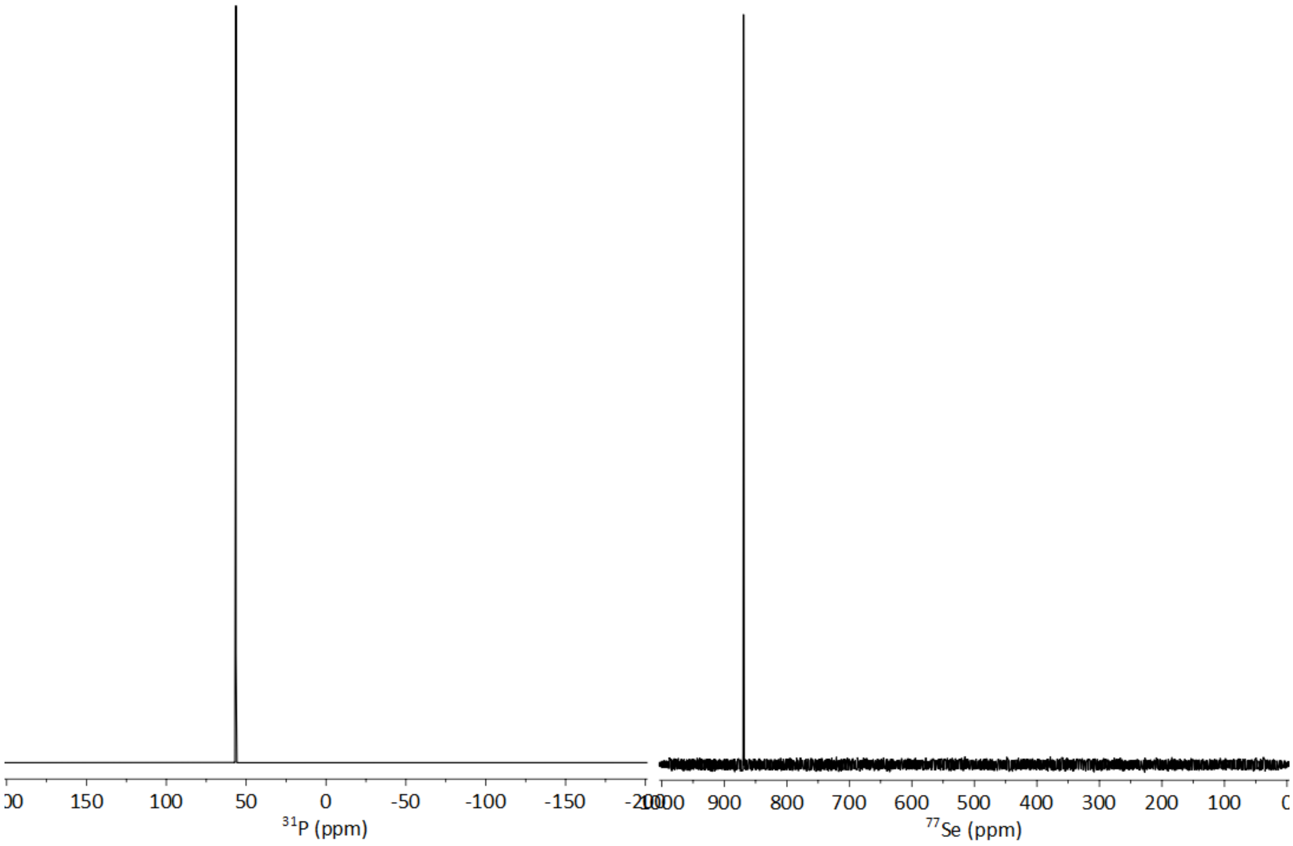

Figure S5. The ^{31}P{^{1}H} NMR (left) and 77Se{1H} NMR (right) spectra of **1-O2** (acquired at 202.5 and 95.4 MHz, respectively)

Infrared Spectra

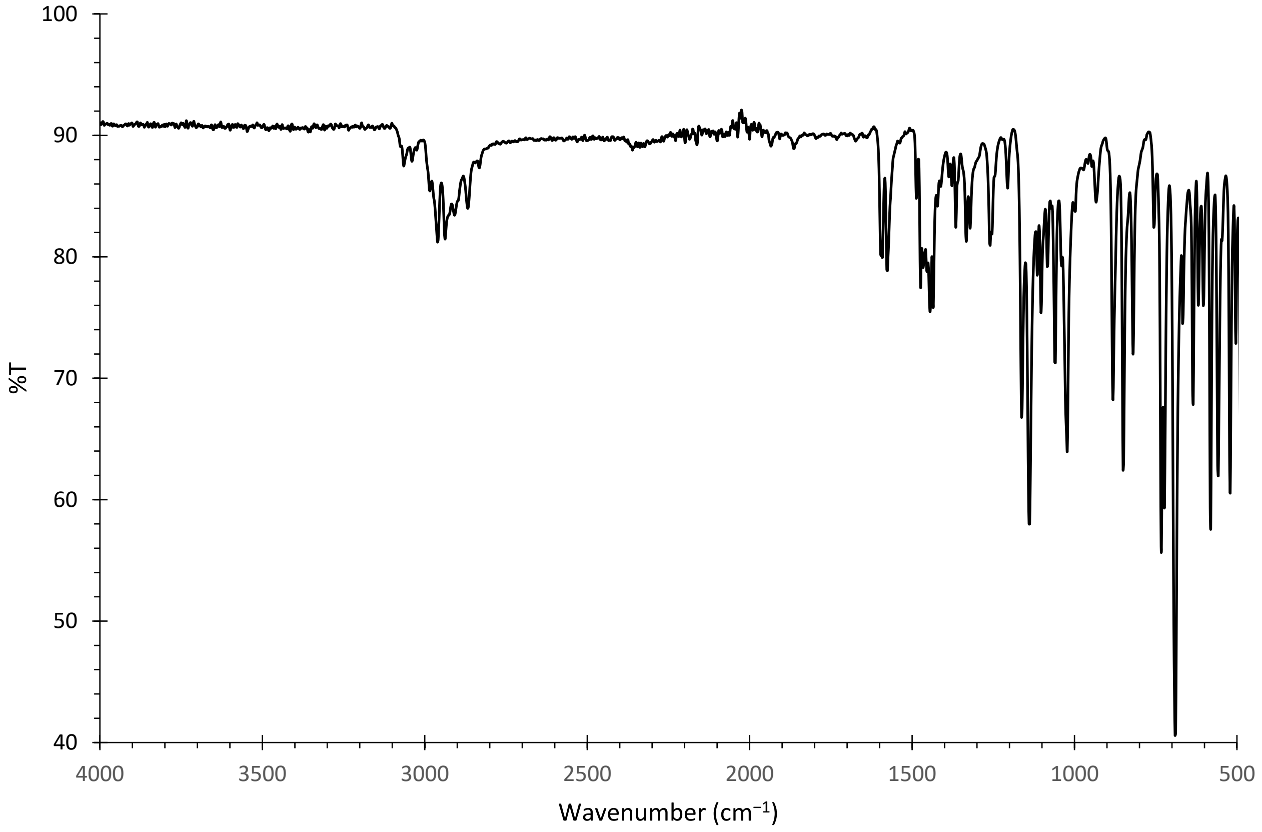

Figure S6. Infrared spectrum of **1-O**.

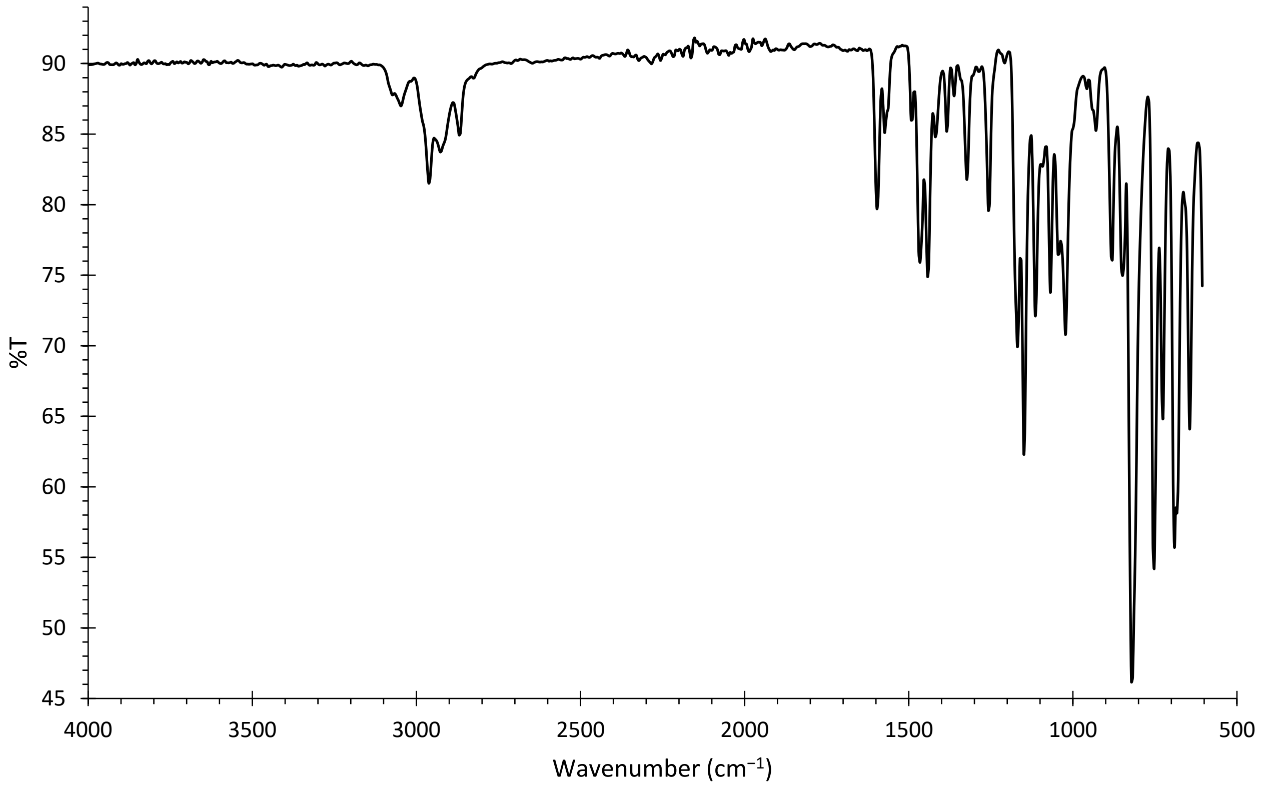

Figure S7. Infrared Spectrum of **1-O2**.

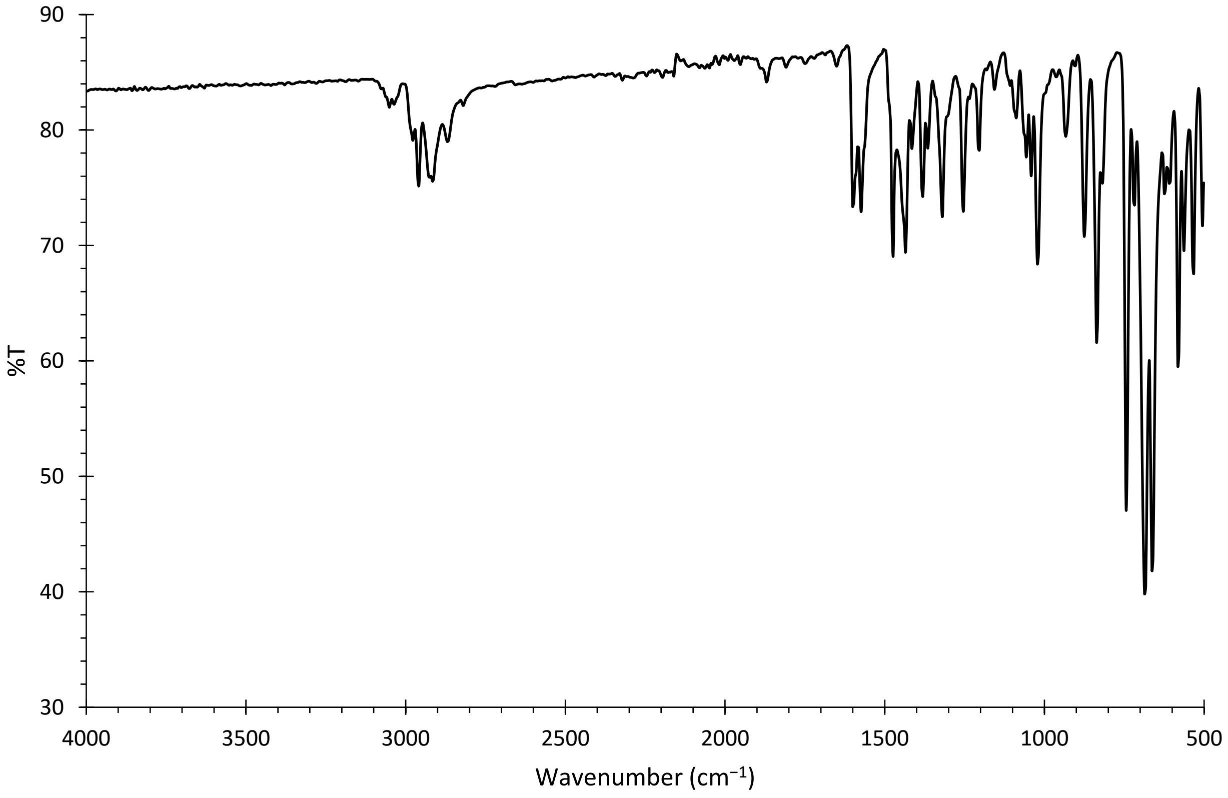

Figure S8. Infrared Spectrum of 1-S.

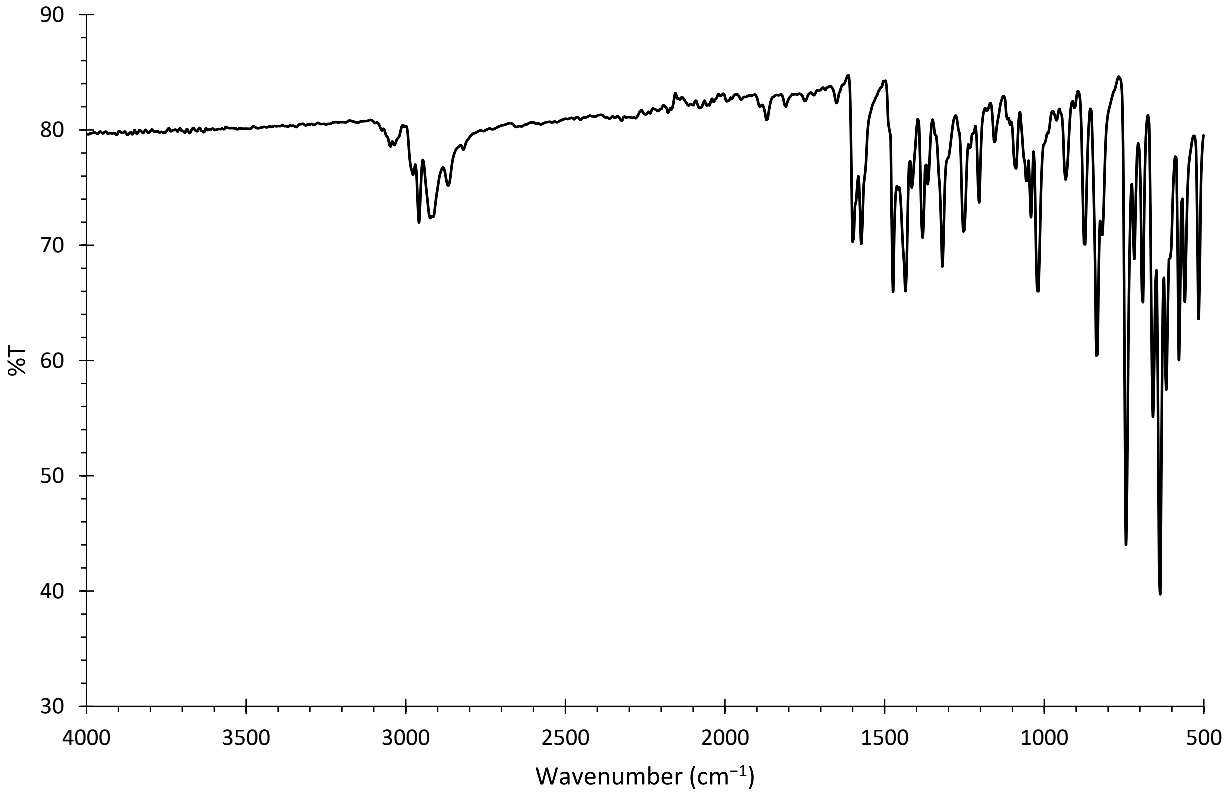

Figure S9. Infrared Spectrum of **1-Se**.

X-ray Diffraction

Table S1 lists details of data collections and refinements.

Table S1. Crystal and Structure Refinement Data.

	1-O	1-O2	1-S	1-Se
**Formula**	$C_{24}H_{27}OPSe$	$C_{24}H_{27}O_2PSe$	$C_{24}H_{27}SPSe$	$C_{24}H_{27}PSe_2$
**M_r**	441.41	457.41	457.47	504.37
**Colour/Habit**	Colourless prism	Colourless prism	Colourless prism	Colourless prism
**Crystal Dimensions [mm]**	0.200×0.200×0.200	0.290×0.120×0.100	0.120×0.090×0.030	0.210×0.110×0.060
**Crystal System**	Monoclinic	Monoclinic	Monoclinic	Monoclinic
**Space Group**	$P2_1/n$ (#14)	$P2_1/n$ (#14)	$P2_1/n$ (#14)	$P2_1/n$ (#14)
***a* [Å]**	8.886(2)	8.2723(3)	9.20428(9)	9.2477(6)
***b* [Å]**	11.950(2)	16.1425(6)	12.20590(11)	12.1871(8)
***c* [Å]**	19.754(4)	32.4731(10)	19.15860(18)	19.0466(13)
**α [°]**	90	90	90	90
**β [°]**	96.223(6)	93.484(3)	97.3870(9)	97.689(5)
**γ [°]**	90	90	90	90
**V [Å^3]**	2085.3(7)	4329.0(3)	2134.54(4)	2127.3(2)
**Z**	4	8	4	4
**$\rho_{calcd.}$ [g cm^{-1}]**	1.406	1.404	1.423	1.575
**μ [cm^{-1}]**	18.888	18.257	40.317	35.587
**$2\theta_{max}$**	50.8	63.7	136.6	50.8
**F_{000}**	912.00	1888.00	944.00	1016.00
**Measured refln.**	24111	53402	21590	18325
**Unique refln.**	3760	14265	3873	3900
**R [I>2σ(I)]**	0.0713	0.0833	0.0233	0.0248
**wR**	0.2117	0.1917	0.0728	0.0563
**Goodness of Fit**	1.252	0.967	1.021	1.006
**Largest peak/hole [e Å^{-3}]**	2.42/−0.79	3.82/−0.98	0.27/−0.40	0.40/−0.29

Computational Details

Cartesian coordinates in Å, B3LYP/6-31+G(d,p) optimised (xyz format)

Compound 1-Se(A)

Se 6.7046103247 2.0185304845 10.6286318189
Se 7.8904596585 1.6533181066 7.5307122048
P 5.8016505397 2.0605307672 7.2106190509
C 6.5893891112 3.8978162205 10.1146005235
C 7.1651237781 4.7792620106 11.0252847985
H 7.7515542945 4.3617890863 11.8374146174
C 6.9829030839 6.1809900497 10.9819810493
H 7.4582872012 6.8103188223 11.7294956338
C 6.1588330645 6.6941026712 10.00835092
C 5.5980525332 5.8136166769 9.0504003058
C 4.7363058128 6.5147004749 8.1710576667
C 4.0858188197 5.8176125724 7.1829831331
H 3.3893261531 6.2935423211 6.4981640162
C 4.3967058006 4.4449322247 7.0248320004
H 3.9447697446 3.9486897844 6.175136027
C 5.2690913237 3.7299594662 7.8451170379
C 5.8376975308 4.4057957218 8.9979257154
C 5.6984853525 8.1130572563 9.7377426833
H 6.5472692513 8.7551343465 9.4739455822
H 5.2291277854 8.5612553965 10.6205952014
C 4.6955865024 7.9819480095 8.5478207066
H 3.6823273587 8.279751906 8.8426795176
H 4.9756084842 8.6205955206 7.7026103604
C 5.4736179207 2.0876168505 5.3491490234
H 4.4105181851 2.317018057 5.2124131643
C 5.7405290895 0.702159176 4.7322628055
H 6.765624761 0.3767150342 4.9331945731
H 5.6070815002 0.7650674837 3.6461247201
H 5.0564925957 -0.0641587507 5.1044452838
C 6.324123655 3.152910095 4.6366443835
H 6.1447638493 4.1627170315 5.0135788028
H 6.0894699328 3.1432327681 3.5657103053
H 7.3883669465 2.9319347562 4.758674828
C 4.5753476678 0.8392586803 7.9716997343
H 4.5311908918 1.2001921248 9.0039115034
C 5.085163077 -0.6077473164 8.0166743337
H 5.0965559685 -1.0771779142 7.0285846514
H 4.4210839568 -1.1991291571 8.6582245219
H 6.0970066188 -0.6595738802 8.4261153955
C 3.1663224821 0.9481336305 7.3643442653
H 2.7867880854 1.9747709911 7.3737955029
H 2.474093387 0.3404385462 7.9586255478
H 3.1265003932 0.5724773387 6.3365184272
C 5.7367233461 2.1673373102 12.3251097555
C 6.2294686078 1.4423055015 13.4188378247
H 7.1642466175 0.8953604475 13.3285945664
C 5.5294262704 1.4300775919 14.6305435214
H 5.922342148 0.8649103267 15.4716009915
C 4.3420229212 2.1537911572 14.7648442071
H 3.8031496082 2.151750812 15.708024958
C 3.8525612371 2.8838233393 13.675569079
H 2.928217772 3.4479915056 13.7697675188
C 4.5386336255 2.8844990216 12.4587751876
H 4.1435033726 3.4460975446 11.6174600462

Compound 1-Se(B)

Se 1.4922955863 -1.0825331432 -0.6380511333
Se -2.02640076 -1.1350878348 -1.9373472859
P -2.0118879421 -0.564710372 0.1351849615
C 1.1543027062 0.8179833393 -0.8626421123
C 2.1392903461 1.5328028475 -1.5404621643
H 2.9215571832 0.9768095103 -2.0468840645
C 2.2071456344 2.9419377363 -1.5603467669
H 2.9957166718 3.4361832699 -2.1214227544
C 1.3110733097 3.6404429134 -0.7837530547
C 0.305427507 2.9278277053 -0.0886128303
C -0.4520759397 3.806349975 0.7270455166
C -1.4627324706 3.2893765732 1.4962610792
H -2.0509863933 3.9024612299 2.1735047486
C -1.7862852083 1.9197129519 1.3306463477
H -2.6603064138 1.5731721455 1.8661419409
C -1.1033807895 1.0332345463 0.49845441
C 0.1016864817 1.5130141178 -0.1670398279
C 1.1937157549 5.1232303941 -0.4917438955
H 0.9182798014 5.6775385433 -1.3971954877
H 2.1421302846 5.5456477063 -0.1418255106
C 0.0739963933 5.2208811048 0.5916476489
H 0.4748101189 5.5772122781 1.5482119581
H -0.7190265456 5.92010543 0.3043937576
C -3.8098101265 -0.2762695697 0.6576189824
H -3.7992817754 0.0289395622 1.7098716922
C -4.6153542957 -1.5854869854 0.5559709438
H -4.5702206248 -1.9897455521 -0.4595076397
H -5.6638035901 -1.3747235213 0.7966801023
H -4.2665248089 -2.3537825763 1.2498294864
C -4.4874571959 0.8131811138 -0.1913883919
H -3.9701631122 1.7743885448 -0.1472420559
H -5.5129704722 0.9613553547 0.1672260751
H -4.5282503282 0.5016932373 -1.2388983159
C -1.2988482856 -1.8089951398 1.3731045736
H -0.2312413983 -1.5765608493 1.3379413718
C -1.4582744549 -3.2721407157 0.9392366591
H -2.4957325828 -3.6131510568 0.9977538908
H -0.8595387611 -3.9096105643 1.6009130869
H -1.1160952059 -3.4174488082 -0.0883027106
C -1.7736472153 -1.5604785564 2.8156111547
H -1.6257851068 -0.5216093897 3.1279130748
H -1.189618603 -2.1905198404 3.496574904
H -2.8271692032 -1.8199799911 2.958489227
C 2.1065031467 -1.5903185575 -2.4001819277
C 1.4064222528 -1.2444110223 -3.5636395697
H 0.5040103757 -0.6468281128 -3.4943134492
C 1.8700306331 -1.6904384596 -4.8036120039
H 1.3270371435 -1.4176870345 -5.7045177113
C 3.0108938519 -2.4963185178 -4.8894014247
H 3.3608359243 -2.8454625898 -5.8567399836
C 3.6981156483 -2.849353124 -3.725104126
H 4.5878716344 -3.4705546118 -3.7815397999
C 3.2555128511 -2.3888237691 -2.4806851437
H 3.8063788176 -2.6409240056 -1.5790458023

Compound 1-Se(C)

Se 1.1594762357 -1.340677088 -1.1085236428

Se -1.2758028773 -1.9238003893 1.8666104698
P -1.9546588085 -0.4489404497 0.4530669448
C 1.1779672509 0.6074896054 -1.0936378566
C 2.2235994601 1.1747214573 -1.8162396788
H 2.8482262303 0.5122520405 -2.4065379371
C 2.5405143233 2.5515421932 -1.7860362717
H 3.3730668208 2.9310649761 -2.3721184569
C 1.7968763666 3.3583110565 -0.9590691418
C 0.7159579792 2.7993634539 -0.2307974224
C 0.1416607478 3.7741586232 0.6203406948
C -0.892285667 3.4015698322 1.4412670836
H -1.3449468952 4.0850165904 2.1543532637
C -1.3998848053 2.0885743856 1.3158967316
H -2.2403159459 1.8490717016 1.9515728746
C -0.9105166476 1.1135626415 0.4433799266
C 0.2957343029 1.4304347602 -0.3075127183
C 1.9308575799 4.8342495854 -0.6404778932
H 1.7407450276 5.4448825823 -1.5312530785
H 2.9431142498 5.0813093497 -0.3019624958
C 0.8641518421 5.0963474055 0.4674791274
H 1.3325974834 5.3943887428 1.4128960997
H 0.1741521356 5.9020105366 0.1925387997
C -2.4076385827 -1.0243921879 -1.3165299597
H -3.4907646045 -0.8708494665 -1.294114808
C -2.1949594545 -2.5273321064 -1.5438892708
H -1.1373487627 -2.7885459224 -1.5961126589
H -2.669336451 -2.8102498841 -2.4918719338
H -2.6390013293 -3.122677922 -0.7426839335
C -1.8835525773 -0.1622706881 -2.4761373434
H -2.0117698188 0.9086143913 -2.2936429483
H -2.4471116843 -0.4159953768 -3.3822626548
H -0.8290958498 -0.3417173823 -2.6941445673
C -3.6852010218 0.127145154 1.0110051043
H -3.5284691316 0.4974912043 2.0288236625
C -4.6369958528 -1.0771159607 1.1423832908
H -4.8886812834 -1.5174030041 0.1709913012
H -5.5751348494 -0.7369880699 1.5965519075
H -4.2073750925 -1.8560281659 1.7757715098
C -4.3273438702 1.2341868536 0.1541345292
H -3.6908588034 2.1144868786 0.0376018275
H -5.2620351291 1.5580589956 0.6274178764
H -4.5877474399 0.8726800994 -0.8466294431
C 2.6693045253 -1.6562808272 0.0764857863
C 3.8425445601 -2.2062171034 -0.4555492428
H 3.909187736 -2.415556216 -1.5189619127
C 4.9267834596 -2.4802138228 0.3852518438
H 5.8347924808 -2.9090349939 -0.0301355691
C 4.8433483009 -2.1965987776 1.7511391044
H 5.6871213845 -2.4054098041 2.4030144526
C 3.6675926935 -1.6519203492 2.2783125309
H 3.5923505806 -1.443840991 3.3421975173
C 2.5740214441 -1.3895754493 1.448224182
H 1.6460900538 -1.0102131483 1.8635540471

Compound 1-Se(D)

Se 1.6535162581 -1.1994913119 -1.2757243099
Se -3.812282656 -0.6207666557 0.9233705937
P -1.7953483066 -0.7403062305 0.1560570316
C 1.3547157294 0.7134288078 -1.1018213235

C 2.3191136665 1.4588955893 -1.7793756962
H 3.0384853141 0.9202726137 -2.387515261
C 2.4376140595 2.8621775286 -1.6820932026
H 3.211096278 3.3832208028 -2.2391452045
C 1.5793618832 3.5125936822 -0.8281377711
C 0.5778480477 2.7750029049 -0.1470052977
C -0.1571827197 3.6292453189 0.7138077195
C -1.1824079602 3.0996152807 1.4540878491
H -1.7700384803 3.6971366315 2.1455280801
C -1.5179911214 1.7433923223 1.2439642238
H -2.4079262201 1.365517209 1.7387700179
C -0.8373944746 0.8710180784 0.3963945068
C 0.3467956263 1.3648481896 -0.2926253385
C 1.4945419282 4.9782235489 -0.4536567114
H 1.2300119991 5.5844569943 -1.3282647646
H 2.4561191276 5.3570667659 -0.0901577597
C 0.3847376029 5.0415758403 0.6391670221
H 0.7939751974 5.3510417408 1.6082285095
H -0.4019292944 5.7612028934 0.3876181594
C -0.8760441437 -2.1355496277 1.035329316
H -0.0363785368 -2.3859844876 0.3849403162
C -1.7499945867 -3.3830592255 1.2422800397
H -2.6061381172 -3.1593353897 1.8841642144
H -1.1411106413 -4.1572995924 1.724996359
H -2.1351637522 -3.7938996202 0.3065664838
C -0.3303937506 -1.6320018029 2.382964483
H 0.3898660749 -0.8191374982 2.2630675092
H 0.1785779814 -2.4570505947 2.8937430409
H -1.142658335 -1.2847175888 3.0308073907
C -1.829066524 -0.9699888709 -1.7110267868
H -0.7960344361 -0.8472713595 -2.0452739783
C -2.6788162323 0.1354662862 -2.3600755185
H -3.7255916748 0.0520030007 -2.055416026
H -2.6200948443 0.03598575 -3.4503444552
H -2.3269666566 1.1364439549 -2.0926447159
C -2.3124313473 -2.366009834 -2.1292901144
H -1.6283736671 -3.1547209389 -1.8030863089
H -2.3717406588 -2.412762973 -3.222930959
H -3.3086973284 -2.5751044553 -1.7272733814
C 2.9372438432 -1.5062212428 0.1502176935
C 3.4122278331 -2.8196822492 0.281497075
H 3.0694590885 -3.5981210411 -0.3958828056
C 4.3328815885 -3.1329628241 1.2837967453
H 4.69516668 -4.1531751557 1.3760476487
C 4.7854923527 -2.1432280603 2.1622891809
H 5.500310336 -2.3881962663 2.9421623527
C 4.3096182685 -0.8367091932 2.0269489129
H 4.6527554899 -0.0590601554 2.7040248749
C 3.388065662 -0.5123145022 1.0256092
H 3.0289001599 0.5068079428 0.9341032106
