# Supplementary material for: Constrained Phosphine Chalcogenide Selenoethers Supported by peri-Substitution
Source: Molecules. 2023 Oct 27;28(21):7297. doi: 10.3390/molecules28217297 (PMC10650533; doi:10.3390/molecules28217297)

checkCIF/PLATON report

Structure factors have been supplied for datablock(s) 1-O2

THIS REPORT IS FOR GUIDANCE ONLY. IF USED AS PART OF A REVIEW PROCEDURE FOR PUBLICATION, IT SHOULD NOT REPLACE THE EXPERTISE OF AN EXPERIENCED CRYSTALLOGRAPHIC REFEREE.

No syntax errors found.   CIF dictionary   Interpreting this report

Datablock: 1-O2

Bond precision:	C-C = 0.0079 A		Wavelength=0.71073
Cell:	a=8.2736(3)	b=16.1425(6)	c=32.4731(10)
	alpha=90	beta=93.484(3)	gamma=90
Temperature:	173 K		

	Calculated	Reported
Volume	4329.0(3)	4329.0(3)
Space group	P 21/n	P 1 21/n 1
Hall group	-P 2yn	-P 2yn
Moiety formula	C24 H27 O2 P Se	C24 H27 O2 P Se
Sum formula	C24 H27 O2 P Se	C24 H27 O2 P Se
Mr	457.39	457.41
Dx,g cm-3	1.404	1.404
Z	8	8
Mu (mm-1)	1.825	1.826
F000	1888.0	1888.0
F000'	1888.70	
h,k,lmax	12,23,48	12,23,47
Nref	14848	14265
Tmin,Tmax	0.769,0.833	0.691,0.833
Tmin'	0.589	

Correction method= # Reported T Limits: Tmin=0.691 Tmax=0.833
AbsCorr = MULTI-SCAN

Data completeness= 0.961   Theta(max)= 31.865

R(reflections)= 0.0833(8957)   wR2(reflections)= 0.1917(14265)

S = 0.967   Npar= 513

The following ALERTS were generated. Each ALERT has the format
test-name_ALERT_alert-type_alert-level.
Click on the hyperlinks for more details of the test.

Alert level A

```
PLAT971_ALERT_2_A Check Calcd Resid. Dens.  1.28Ang From Se1            3.55 eA-3
```

Author Response: Crystal quality - does not affect crystallorapich results.

Alert level B

```
PLAT097_ALERT_2_B Large Reported Max.  (Positive) Residual Density       3.82 eA-3
```

Author Response: Crystal quality - does not affect crystallorapich results.

Alert level C

```
DIFMX02_ALERT_1_C  The maximum difference density is > 0.1*ZMAX*0.75
            The relevant atom site should be identified.
PLAT094_ALERT_2_C Ratio of Maximum / Minimum Residual Density ....       3.90 Report
PLAT242_ALERT_2_C Low    'MainMol' Ueq as Compared to Neighbors of        C13 Check
PLAT341_ALERT_3_C Low Bond Precision on  C-C Bonds ..............     0.0079 Ang.
PLAT413_ALERT_2_C Short Inter XH3 .. XHn     H15A     ..H41A     .       2.09 Ang.
                                            1-x,1-y,1-z  =      3_666 Check
PLAT906_ALERT_3_C Large K Value in the Analysis of Variance ......     11.564 Check
PLAT906_ALERT_3_C Large K Value in the Analysis of Variance ......      3.055 Check
PLAT911_ALERT_3_C Missing FCF Refl Between Thmin & STh/L=    0.600          7 Report
```

Alert level G

```
PLAT083_ALERT_2_G SHELXL Second Parameter in WGHT  Unusually Large      33.63 Why ?
PLAT910_ALERT_3_G Missing # of FCF Reflection(s) Below Theta(Min).         2 Note
PLAT912_ALERT_4_G Missing # of FCF Reflections Above STh/L=  0.600       571 Note
PLAT913_ALERT_3_G Missing # of Very Strong Reflections in FCF ....         3 Note
PLAT941_ALERT_3_G Average HKL Measurement Multiplicity ..........       3.7 Low
PLAT978_ALERT_2_G Number C-C Bonds with Positive Residual Density.         3 Info
```

1 **ALERT level A** = Most likely a serious problem - resolve or explain
1 **ALERT level B** = A potentially serious problem, consider carefully
8 **ALERT level C** = Check. Ensure it is not caused by an omission or oversight
6 **ALERT level G** = General information/check it is not something unexpected

1 ALERT type 1 CIF construction/syntax error, inconsistent or missing data
7 ALERT type 2 Indicator that the structure model may be wrong or deficient
7 ALERT type 3 Indicator that the structure quality may be low
1 ALERT type 4 Improvement, methodology, query or suggestion
0 ALERT type 5 Informative message, check

It is advisable to attempt to resolve as many as possible of the alerts in all categories. Often the minor alerts point to easily fixed oversights, errors and omissions in your CIF or refinement strategy, so attention to these fine details can be worthwhile. In order to resolve some of the more serious problems it may be necessary to carry out additional measurements or structure refinements. However, the purpose of your study may justify the reported deviations and the more serious of these should normally be commented upon in the discussion or experimental section of a paper or in the "special_details" fields of the CIF. checkCIF was carefully designed to identify outliers and unusual parameters, but every test has its limitations and alerts that are not important in a particular case may appear. Conversely, the absence of alerts does not guarantee there are no aspects of the results needing attention. It is up to the individual to critically assess their own results and, if necessary, seek expert advice.

Publication of your CIF in IUCr journals

A basic structural check has been run on your CIF. These basic checks will be run on all CIFs submitted for publication in IUCr journals (*Acta Crystallographica*, *Journal of Applied Crystallography*, *Journal of Synchrotron Radiation*); however, if you intend to submit to *Acta Crystallographica Section C* or *E* or *IUCrData*, you should make sure that full publication checks are run on the final version of your CIF prior to submission.

Publication of your CIF in other journals

Please refer to the *Notes for Authors* of the relevant journal for any special instructions relating to CIF submission.

PLATON version of 10/05/2023; check.def file version of 10/05/2023

Datablock 1-O2 - ellipsoid plot

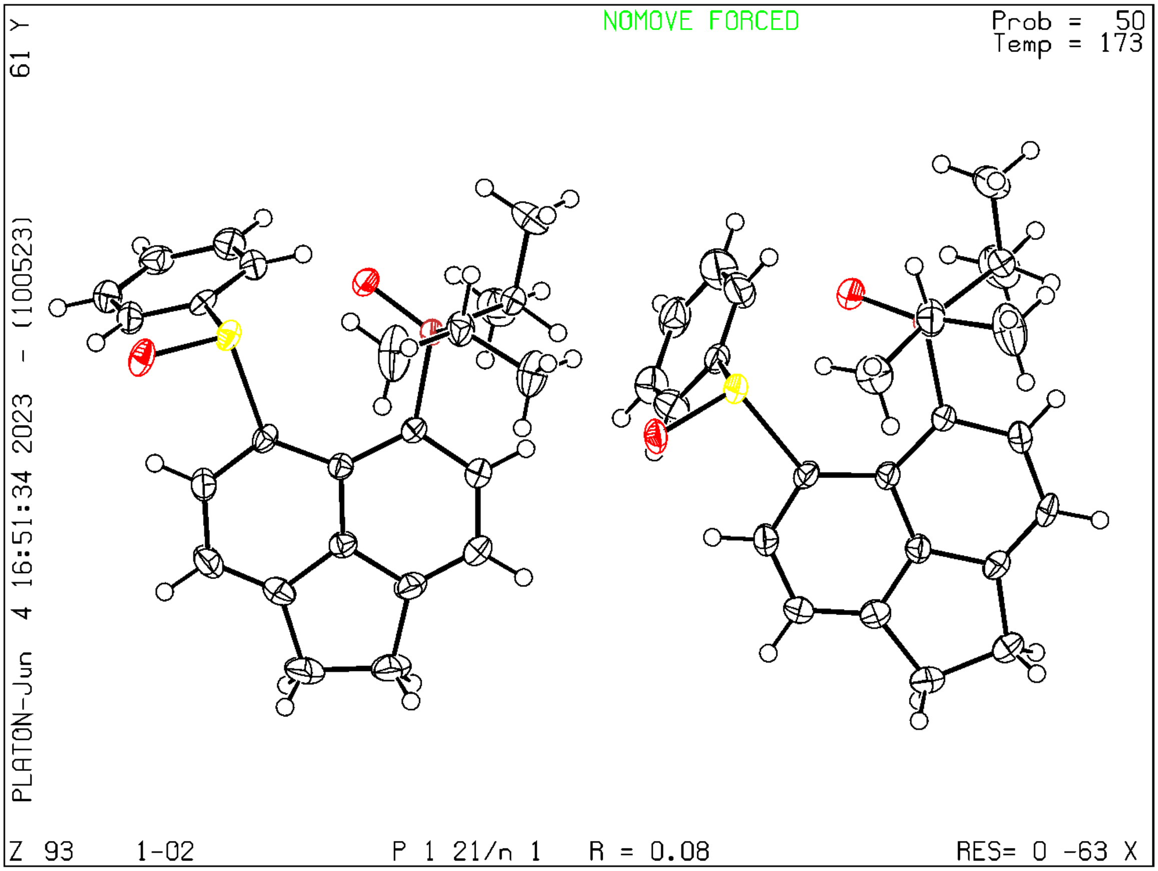

Supplement: Supplementary file 1 [file 1-O2checkcif.pdf › Supporting Information/SC XRD/1-O2checkcif.pdf]
